# Entomopathogenic Fungus-Related Priming Defense Mechanisms in Cucurbits Impact Spodoptera littoralis (Boisduval) Fitness

**DOI:** 10.1128/aem.00940-23

**Published:** 2023-07-13

**Authors:** F. García-Espinoza, M. J. García, E. Quesada-Moraga, M. Yousef-Yousef

**Affiliations:** a Departamento de Agronomía (DAUCO) María de Maeztu Unit of Excellence 2021–2023, Campus de Rabanales, Universidad de Córdoba, Córdoba, Spain; b Departamento de Parasitología. Universidad Autónoma Agraria Antonio Narro – Unidad Laguna, Torreón, Coahuila, Mexico; Royal Botanic Gardens

**Keywords:** *Metarhizium brunneum*, EAMa 01/58-Su strain, cucumber, melon, ISR, Fe deficiency

## Abstract

Entomopathogenic fungi (EPF) exhibit direct and indirect mechanisms to increase plant resistance against biotic and abiotic stresses. Plant responses to these stresses are interconnected by common regulators such as ethylene (ET), which is involved in both iron (Fe) deficiency and induced systemic resistance responses. In this work, the roots of cucurbit seedlings were primed with Metarhizium brunneum (EAMa 01/58-Su strain), and relative expression levels of 18 genes related to ethylene (ET), jasmonic acid (JA), and salicylic acid (SA) synthesis, as well as pathogen-related (PR) protein genes, were studied by reverse transcription-quantitative PCR (qRT-PCR). Effects of priming on Spodoptera littoralis were studied by feeding larvae for 15 days with primed and control plants. Genes showed upregulation in studied species; however, the highest relative expression was observed in roots and shoots of plants with Fe deficiency, demonstrating the complexity and the overlapping degree of the regulatory network. *EIN2* and *EIN3* should be highlighted; both are key genes of the ET transduction pathway that enhanced their expression levels up to eight and four times, respectively, in shoots of primed cucumber. Also, JA and SA synthesis and PR genes showed significant upregulation during the observation period (e.g., the JA gene *LOX1* increased 506 times). Survival and fitness of *S. littoralis* were affected with significant effects on mortality of larvae fed on primed plants versus controls, length of the larval stage, pupal weight, and the percentage of abnormal pupae. These results highlight the role of the EAMa 01/58-Su strain in the induction of resistance, which could be translated into direct benefits for plant development.

**IMPORTANCE** Entomopathogenic fungi are multipurpose microorganisms with direct and indirect effects on insect pests. Also, EPF provide multiple benefits to plants by solubilizing minerals and facilitating nutrient acquisition. A very interesting and novel effect of these fungi is the enhancement of plant defense systems by inducing systematic and acquired resistance. However, little is known about this function. This study sheds light on the molecular mechanisms involved in cucurbits plants’ defense activation after being primed by the EPF *M. brunneum*. Furthermore, the subsequent effects on the fitness of the lepidopteran pest *S. littoralis* are shown. In this regard, a significant upregulation was recorded for the genes that regulate JA, SA, and ET pathways. This increased expression of defense genes caused lethal and sublethal effects on *S. littoralis*. This could be considered an added value for the implementation of EPF in integrated pest management programs.

## INTRODUCTION

Entomopathogenic fungi (EPF) are recognized as excellent biocontrol agents to form part of any integrated pest management (IPM) program due to their capacity to infect a wide range of arthropod pests in a unique way of action, by contact (1–7). Also, they can interact with crops and establish mutualistic interactions that not only protect them against arthropod pests but could also bring benefits to the plant such as plant nutrient acquisition improvement, enhancement of growth and development, immunity, and resistance to other biotic and abiotic stresses (8–19). These functions of EPF led to several multitrophic interactions with important roles in biocontrol (20). Indeed, most EPF species are an important component of the soil microbiota and widely used to control soil-dwelling insect pests, and even well-known rhizosphere-competent microorganism, especially Metarhizium spp. (4, 21–25). In addition to the ability of most EPF to endophytically colonize plant tissues, several species have been shown to provide a systemic protection to the plant by the activation of induced resistance (26–28). This indirect effect of EPF has been poorly studied compared to other, nonentomopathogenic microorganisms with a proven ability to confer resistance on plants, for which several references can be found in the literature, such as bacteria (29–32), mycorrhizae (33–35), and especially the genus *Trichoderma* (36–41), where this indirect effect has been widely studied. In the case of EPF, although some cases of induction of the expression of several genes related to induced resistance have been described, the lethal and sublethal effects shown in these works have been ascribed to the fungus presence in the plant tissues (26, 42); in the case of the Metarhizium genus, the works that can be found in the literature about the induction of systemic resistance are too scarce (42, 43).

Induced resistance refers to the phenomenon that occurs when susceptible plants, as the result of a primary infection by a microbial pathogen, or attack by herbivores or by the interaction with parasitic or nonpathogenic microorganisms, develop defense responses or enhanced genetically programmed resistance to further attack (44–47). Some studies reported upregulation of ethylene (ET), jasmonic acid (JA), salicylic acid (SA), and pathogen-related (PR) genes as endogenous responses of resistant genotypes against phytopathogens such as Phytophthora capsici and Phytophthora melonis (48, 49) or as a result of inoculation/interaction with other microorganisms like bacteria or mycorrhizal fungi (32, 50). Recently, several works have been published that show the effects of EPF on the enhancement of plant defense systems and their lethal and sublethal effects on some pests by direct contact with the fungus strain or by feeding on endophytically colonized tissues (2, 26, 42).

Induced resistance is classified into two types, namely, induced systemic resistance (ISR) and systemic acquired resistance (SAR); SAR is triggered by plant pathogens, and ISR is triggered by root-colonizing mutualistic microbes, generally inhabitants of the rhizosphere (19, 51–54); likewise, when plants are exposed to nonpathogenic microbes, SAR also can be induced (19). Pathogen infection is sensed by innate immune receptors. The binding of conserved microbial molecules (pathogen-associated molecular patterns [PAMPs]) by immune receptors induces PAMP-triggered immunity (PTI), which provides early protection. As a consequence of the coevolution of host and pathogen, PTI is suppressed by pathogen-derived virulence factors (effectors) which are released to host cells to facilitate infection. The recognition of specific pathogen effectors by intracellular nucleotide-binding/leucine-rich-repeat (NLR) receptors activates the effector-triggered immunity (ETI). ETI induces PTI-associated defense pathways, including the production of reactive oxygen species (ROS), mobilization of Ca^2+^-dependent protein kinase and mitogen-activated protein kinase (MAPK) signaling cascades, generation of the phenolic hormone SA, and transcriptional reprogramming (55).

ISR responses are mainly regulated by ET and JA and typically independent of SA and function without PR gene activation (45, 47, 49, 51, 52, 56–59). In contrast, SAR is associated with pathogen infection, and it is characterized by increased SA levels which, through the redox-regulated protein non-expressor of PR genes 1 (NPR1), activate the expression of a large set of PR genes involved in defense responses (47, 51, 52, 57, 58, 60, 61). SA accumulation can be controlled by some protein regulators, such as enhanced disease susceptibility 1 (EDS1), phytoalexin-deficient 4 (PAD4), EDS4, EDS5, and non-race-specific disease resistance 1 (NDR1); likewise, SA can enhance the expression of EDS1/PAD4/SAG101 through a positive-feedback loop (54).

The cross-communication between these hormone signals permits the plant to finely balance the defense response (62). ET and JA act in an antagonistic way to regulate plant responses against cold, drought, and salinity stress (63, 64); however, against necrotrophic fungi and wounds, ET and JA act synergistically to coordinate plant defense responses (54, 65). SA inhibits the JA/ET pathway by the activation of NPR1. The cross-point between JA and ET signaling pathways occurs at the level of ERF1, an ET response factor. JA can promote the activation of MYC2 transcription factor to induce the JA response signal through the interaction between JAZ, a repressor of JA signaling, and the SCFCOI1 ubiquitin ligase, which results in the ubiquitination of the JAZ protein and its degradation by the 26S proteasome (54). JAZ1 can also interact with DELLA proteins, which result in increased JA signaling and decreased SA (66). The DELLA family proteins are key regulators of GA signaling that repress transcription of GA-responsive genes (67). Low GA hormone levels and high ET levels result in a high abundance of DELLA proteins. An increase in GA hormone levels results in GA binding its receptor GID1, which induces interaction with DELLA proteins. The GA-GID1-DELLA complex interacts with SCFSLY1/GID2, an E3 ubiquitin ligase, targeting it for proteasomal degradation, which results in a decrease of DELLA abundance. This reduction in DELLAs initiates transcription of gibberellin response genes and release of JAZ1, which results in an increase of SA signaling (66).

Induced resistance, including ISR and SAR, is associated with an enhanced ability to resist pathogen attack by stronger activation of cellular defense responses (68–72); this enhanced ability or activation of defense is known as “priming” (46, 68–70, 73, 74). Originally, priming was described as an enhanced resistance in response to natural or synthetic chemical agents (56, 71, 75). Nowadays, priming has been described in response to rhizosphere microbes, EPF, or pathogens (17, 51, 62, 68–70, 76); in this sense, plant defense priming could be used as an integrated pest management strategy for crop protection (27, 28).

Some of these ISR inducer microorganisms also promote plant growth and development (77) and favor Fe acquisition in plants (18, 78–83). This is in part due to the common involvement of ET and nitric oxide (NO) in the regulation of both processes and because of the cross talk among ET and JA signaling pathways (52). The effect of these microorganisms on the improvement of Fe nutrition is related to their ability to upregulate key genes related to Fe acquisition, such as *FIT*, *MYB72*, *IRT1*, and *FRO2* (18, 52, 77, 78). On the other hand, MYB72, a key transcription factor (TF) in ISR activation, also participates in the regulation of the Fe deficiency responses through its interaction with FIT TF, a key regulator of Fe deficiency responses.

Due to this cross talk among Fe deficiency responses and ISR in the present work, we aimed to evaluate the ability of Metarhizium brunneum Petch (Hypocreales: Clavicipitaceae) strain EAMa 01/58-Su to induce defense responses in cucumber and melon plants under two nutritional conditions, Fe sufficiency and deficiency, to highlight the cross talk among biotic and abiotic stresses. Relative expression of several genes involved in the JA, SA, and ET synthesis/signaling pathways as well as the induction of PR protein genes was studied. Furthermore, we evaluated the effect of root priming by the EAMa 01/58-Su strain on the survival and development of larvae of the cotton leafworm, Spodoptera littoralis (Boisduval) (Lepidoptera: Noctuidae), a widely distributed, very dangerous polyphagous insect pest that has been previously demonstrated to be susceptible to this fungal strain either by contact or by feeding on endophytically colonized tissues (2).

## RESULTS

### Genes related to ethylene biosynthesis and transduction pathway.

**FIG 1:**
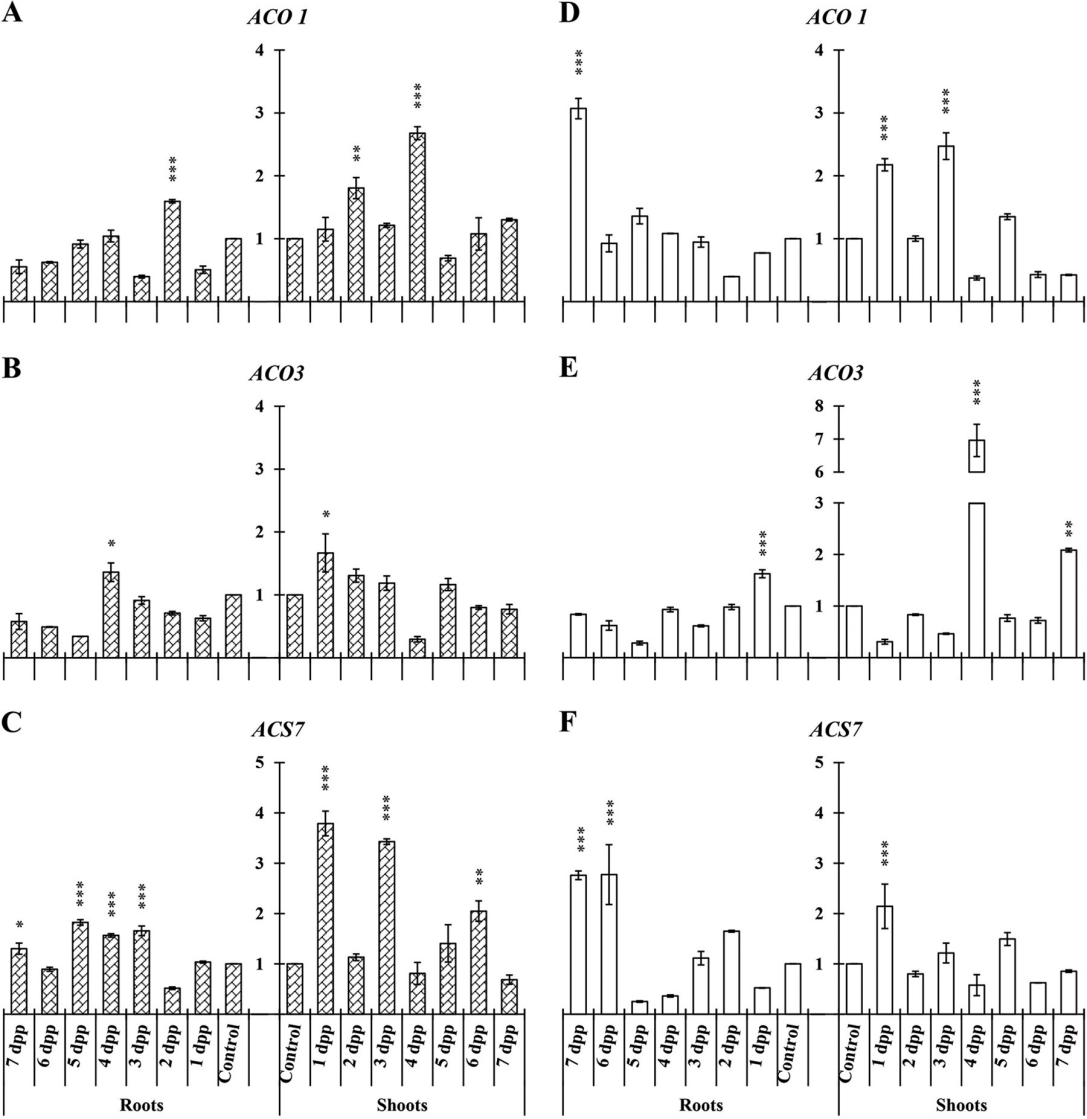
Time course evolution of the relative expression of genes involved in ET biosynthesis on roots and shoots of *C. sativus*. Plants were primed by root immersion during 30 min in an EAMa 01/58-Su solution with 1 × 10^7^ conidia/mL; plants were maintained in a hydroponic system. Samples were collected during 7 days postpriming for qRT-PCR gene expression study. Patterned bars (left) and white bars (right) represent gene expression of plants grown under Fe-sufficient and Fe-deficient conditions, respectively. Data for *ACO1*, *ACO3*, and *ACS7* expression represent the mean from three independent biological replicates ± SE. The relative expression is based on the expression ratio of a target gene versus a reference gene. Bars with *, **, or *** indicate significant differences (*P* < 0.05, *P* < 0.01, or *P* < 0.001, respectively) in relation to their respective control (+Fe40μM or −Fe) according to Dunnett’s test.

In cucumber roots and shoots, three genes related to ET biosynthesis were studied, namely, *ACO1*, *ACO3*, and *ACS7* (Fig. 1). In roots we found significant differences in relative expression levels of the three genes studied under both nutritional conditions at different time points in each case (Fig. 1).

Higher relative expression levels of *ACO1* and *ACS7* could be observed in primed plant shoots under Fe-sufficient conditions (Fig. 1A and C), and under Fe-deficient conditions in the case of *ACO3* (Fig. 1F).

**FIG 2:**
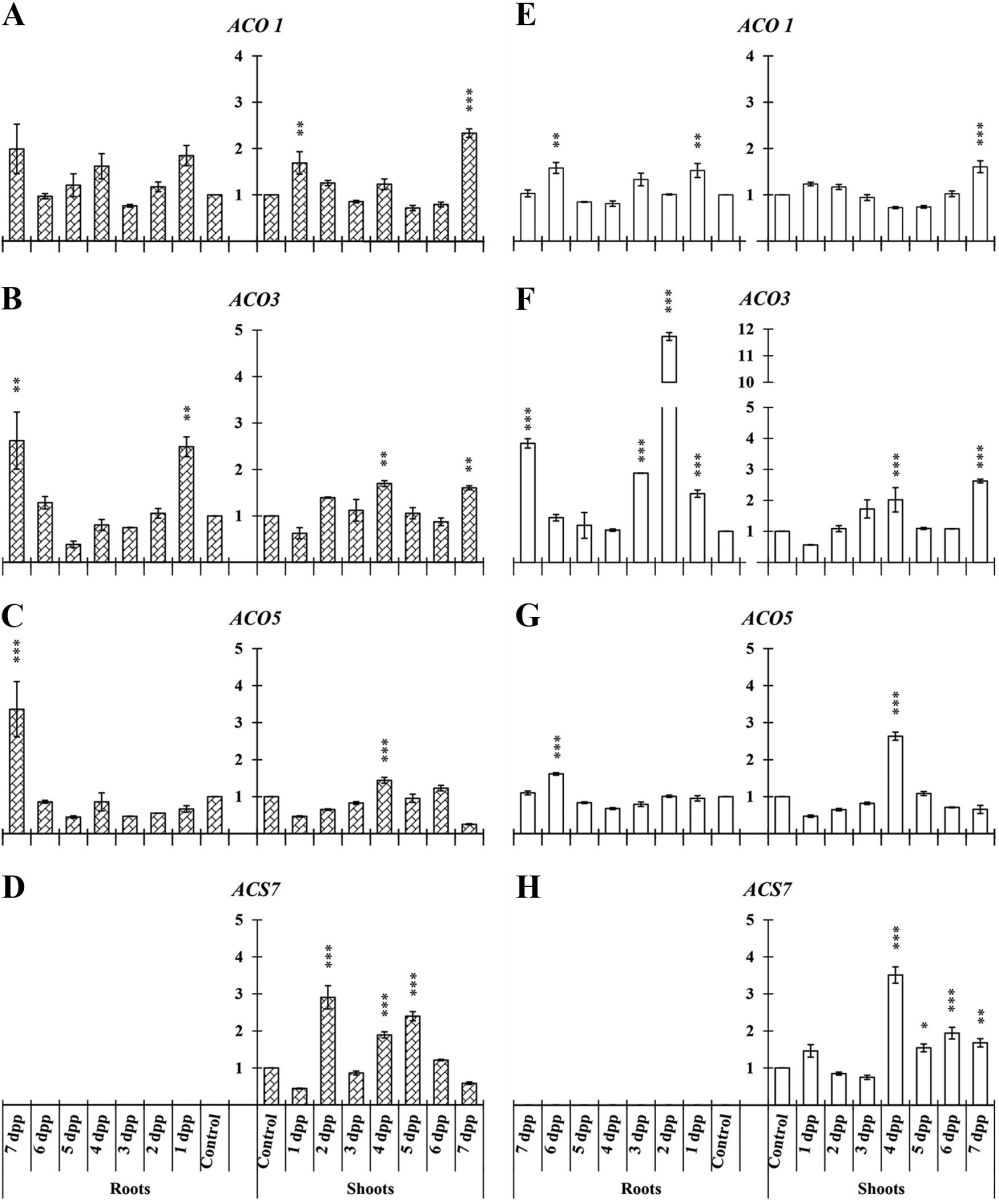
Time course evolution of the relative expression of genes involved in ET biosynthesis on roots and shoots of *C. melo*. Plants were primed by root immersion during 30 min in an EAMa 01/58-Su solution with 1 × 10^7^ conidia/mL; plants were maintained in a hydroponic system. Samples were collected during 7 days postpriming for qRT-PCR gene expression study. Patterned bars (left) and white bars (right) represent gene expression of plants grown under Fe-sufficient and Fe-deficient conditions, respectively. Data for *ACO1*, *ACO3*, *ACO5*, and *ACS7* expression represent the mean from three independent biological replicates ± SE. The relative expression is based on the expression ratio of a target gene versus a reference gene. Bars with *, **, or *** indicate significant differences (*P* < 0.05, *P* < 0.01, or *P* < 0.001, respectively) in relation to their respective control (+Fe40μM or −Fe) according to Dunnett’s test.

In melon, the studied genes related to ET biosynthesis in roots and shoots were the following: *ACO1*, *ACO3*, *ACO5*, and *ACS7.* In roots, only *ACO3* and *ACO5* increased significantly their relative expression under both conditions (Fig. 2B, C, F, and G); significant differences in relative expression levels of *ACO1* in roots of primed plants also could be observed under Fe-deficient conditions (Fig. 2E). *ACS7* was not detected in roots.

In shoots of primed plants all 4 genes studied showed a significant increment of their relative expression under both conditions at different time points (Fig. 2). *ACS7* showed an important increase of its relative expression at different times under both nutritional conditions (Fig. 2D and H).

Besides ET biosynthesis genes, the relative expression of three key genes in the ET transduction pathway was studied in roots and shoots of cucumber (*EIN2* and *EIN3*) and melon (*EIN2*, *EIN3*, and the AP2-like ethylene-responsive transcription factor *MELO3C019787*).

**FIG 3:**
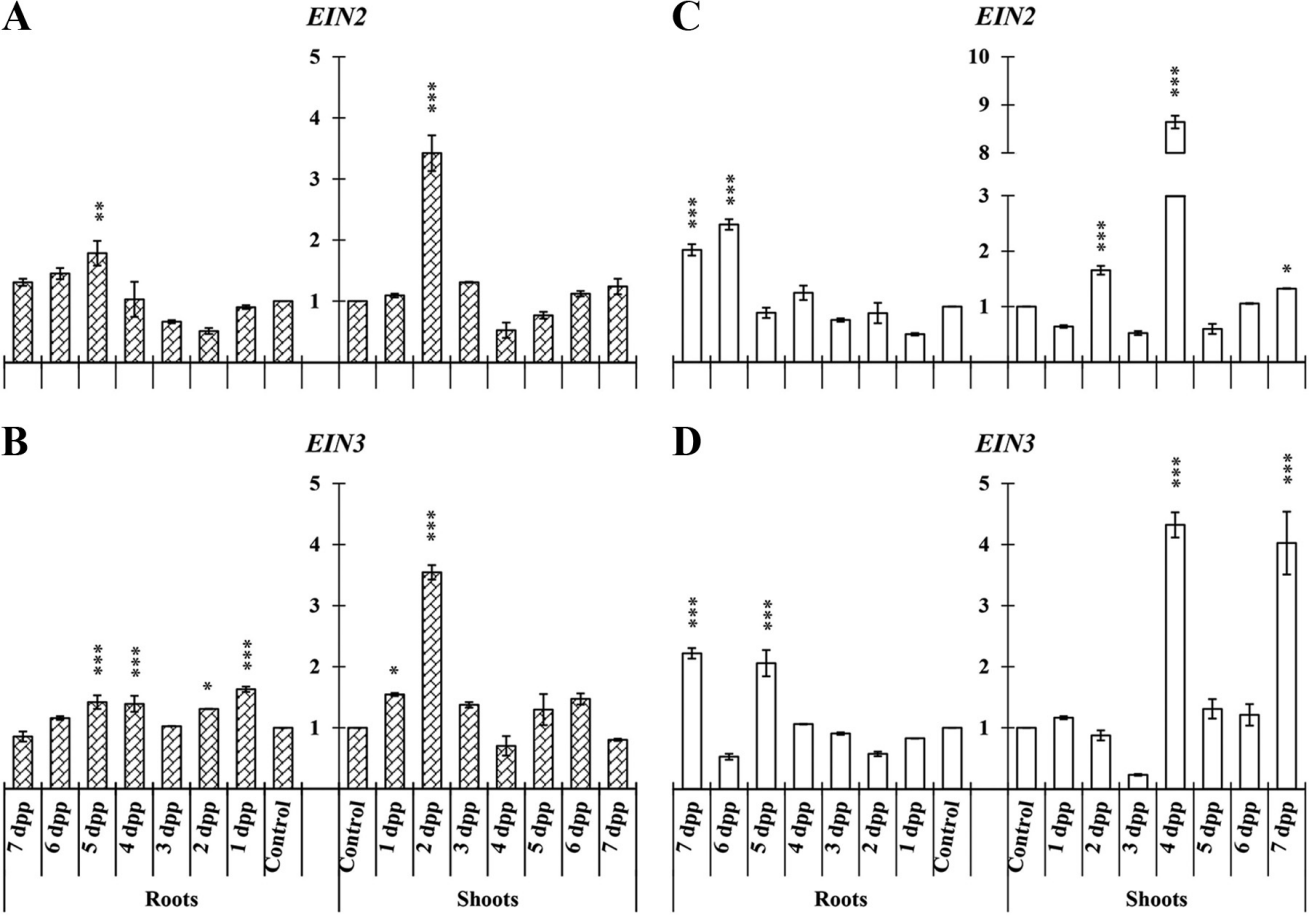
Time course evolution of the relative expression of genes involved in ET transduction pathway on roots and shoots of *C. sativus*. Plants were primed by root immersion during 30 min in an EAMa 01/58-Su solution with 1 × 10^7^ conidia/mL; plants were maintained in a hydroponic system. Samples were collected during 7 days postpriming for qRT-PCR gene expression study. Patterned bars (left) and white bars (right) represent gene expression of plants grown under Fe-sufficient and Fe-deficient conditions, respectively. Data for *EIN2* and *EIN3* expression represent the mean from three independent biological replicates ± SE. The relative expression is based on the expression ratio of a target gene versus a reference gene. Bars with *, **, or *** indicate significant differences (*P* < 0.05, *P* < 0.01, or *P* < 0.001, respectively) in relation to their respective control (+Fe40μM or −Fe) according to Dunnett’s test.

In cucumber *EIN2* and *EIN3* relative expression significantly increased at several time points in roots and shoots under both nutritional conditions (Fig. 3). In shoots of cucumber, the relative expression values reached were higher under both nutritional conditions than the ones observed in roots. For both *EIN2* and *EIN3* genes, an induction peak could be observed at 2 days postpriming (dpp) under Fe-sufficient conditions (Fig. 3A and B) and at 4 dpp under Fe-deficient conditions (Fig. 3C and D).

**FIG 4:**
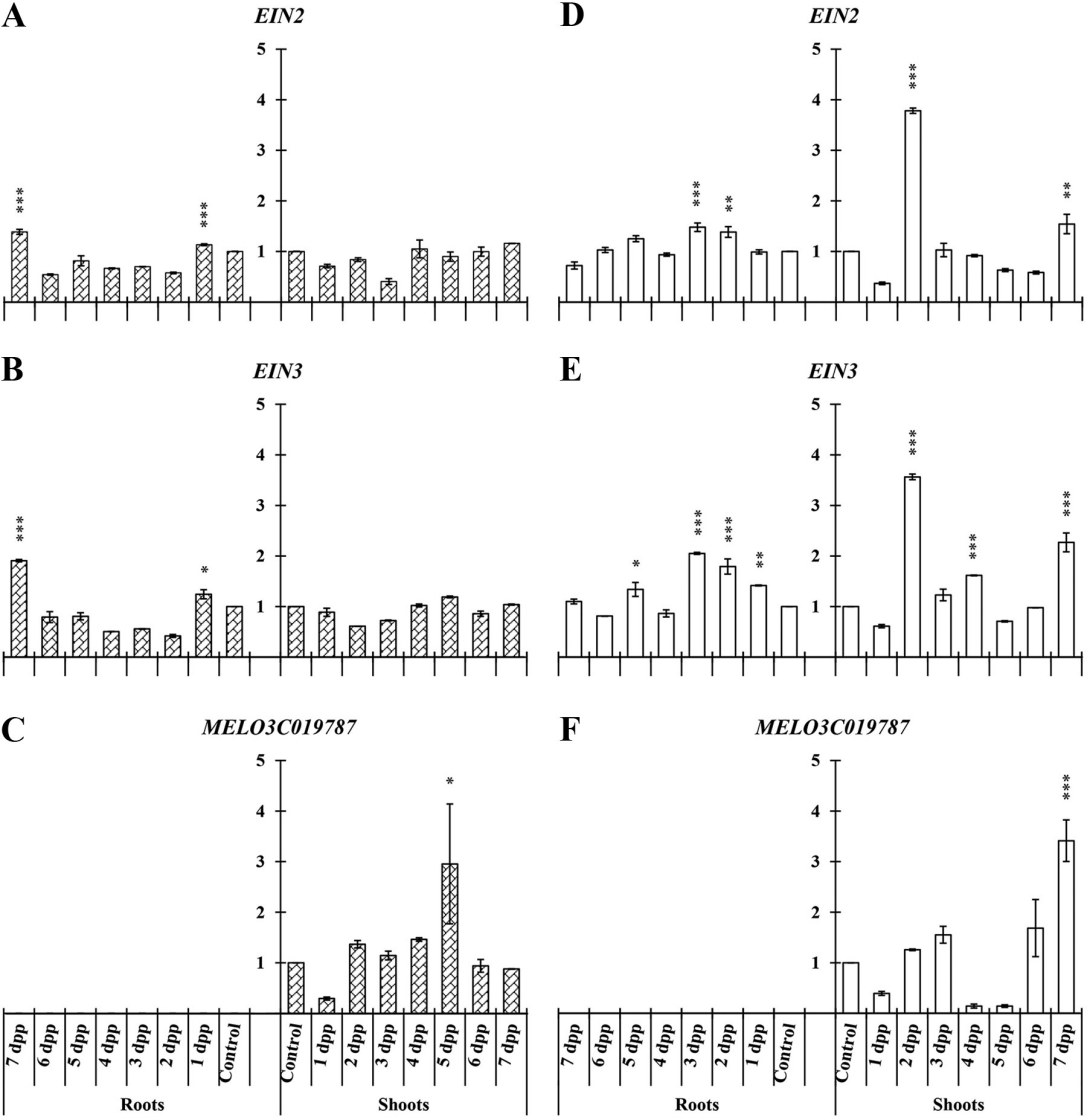
Time course evolution of the relative expression of genes involved in ET transduction (*EIN2* and *EIN3*) and *MELO3C019787* as an ET transcription gene in roots and shoots of *C. melo*. Plants were primed by root immersion during 30 min in an EAMa 01/58-Su solution with 1 × 10^7^ conidia/mL; plants were maintained in a hydroponic system. Samples were collected during 7 days postpriming for qRT-PCR gene expression study. Patterned bars (left) and white bars (right) represent gene expression of plants grown under Fe-sufficient and Fe-deficient conditions, respectively. Data for *EIN2*, *EIN3*, and *MELO3C019787* expression represent the mean for three independent biological replicates ± SE. The relative expression is based on the expression ratio of a target gene to a reference gene. Bars with *, **, or *** indicate significant differences (*P* < 0.05, *P* < 0.01, or *P* < 0.001, respectively) in relation to their respective control (+Fe40μM or −Fe) according to Dunnett’s test.

In melon roots of primed plants, *EIN2* and *EIN3* showed similar relative expression levels under both Fe-sufficient and -deficient conditions (Fig. 4A, B, D, and E). *MELO3C019787* was not detected in roots.

In melon shoots of primed plants both genes enhanced significantly their expression in comparison with their respective controls at 2 and 7 dpp only under Fe-deficient conditions (Fig. 4D and E). Finally, *MELO3C019787* enhanced its relative expression level in shoots only at 5 and 7 dpp under Fe-sufficient and -deficient conditions, respectively (Fig. 4C and E).

### Genes related to JA and SA biosynthesis.

**FIG 5:**
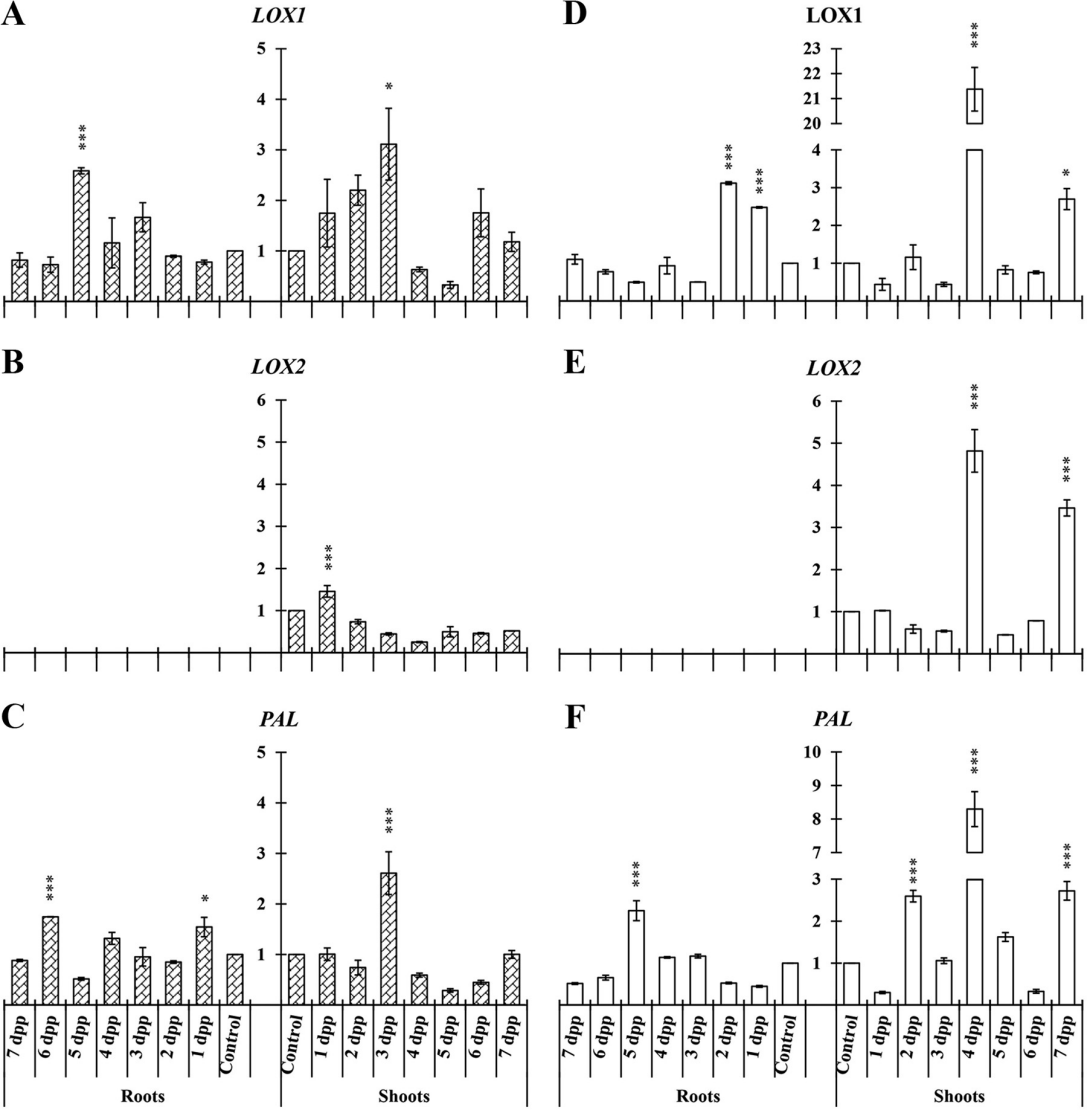
Time course evolution of the relative expression of genes involved in JA (*LOX1* and *LOX2*) and SA (*PAL*) biosynthesis in roots and shoots of *C. sativus*. Plants were primed by root immersion during 30 min in an EAMa 01/58-Su solution with 1 × 10^7^ conidia/mL; plants were maintained in a hydroponic system. Samples were collected during 7 days postpriming for qRT-PCR gene expression study. Patterned bars (left) and white bars (right) represent gene expression of plants grown under Fe-sufficient and Fe-deficient conditions, respectively. Data for *LOX1*, *LOX2*, and *PAL* expression represent the mean from three independent biological replicates ± SE. The relative expression is based on the expression ratio of a target gene to a reference gene. Bars with *, **, or *** indicate significant differences (*P* < 0.05, *P* < 0.01, or *P* < 0.001, respectively) in relation to their respective control (+Fe40μM or −Fe) according to Dunnett’s test.

Relative expression of *LOX1*, *LOX2*, and *PAL* was analyzed in cucumber roots and shoots. In roots *LOX1* and *PAL* showed significant increase of their relative expression under both nutritional conditions (Fig. 5A, C, D, and F). *LOX2* was not detected in roots.

In cucumber shoots of primed plants significant increment of *LOX1*, *LOX2*, and *PAL* expression could be observed under both nutritional conditions at different times. The relative expression increase of these genes in primed plant shoots under Fe-deficient conditions reached its maximum at 4 dpp (Fig. 5D, E, and F).

In the case of melon, as in cucumber, we analyzed two genes related to JA biosynthesis, *LOX2* and *MELO3C014632*, which encodes linoleate 13S-lipoxygenase 2-1, and a gene related to SA biosynthesis, *MELO3C014222*, a phenylalanine ammonia lyase, in roots and shoots.

**FIG 6:**
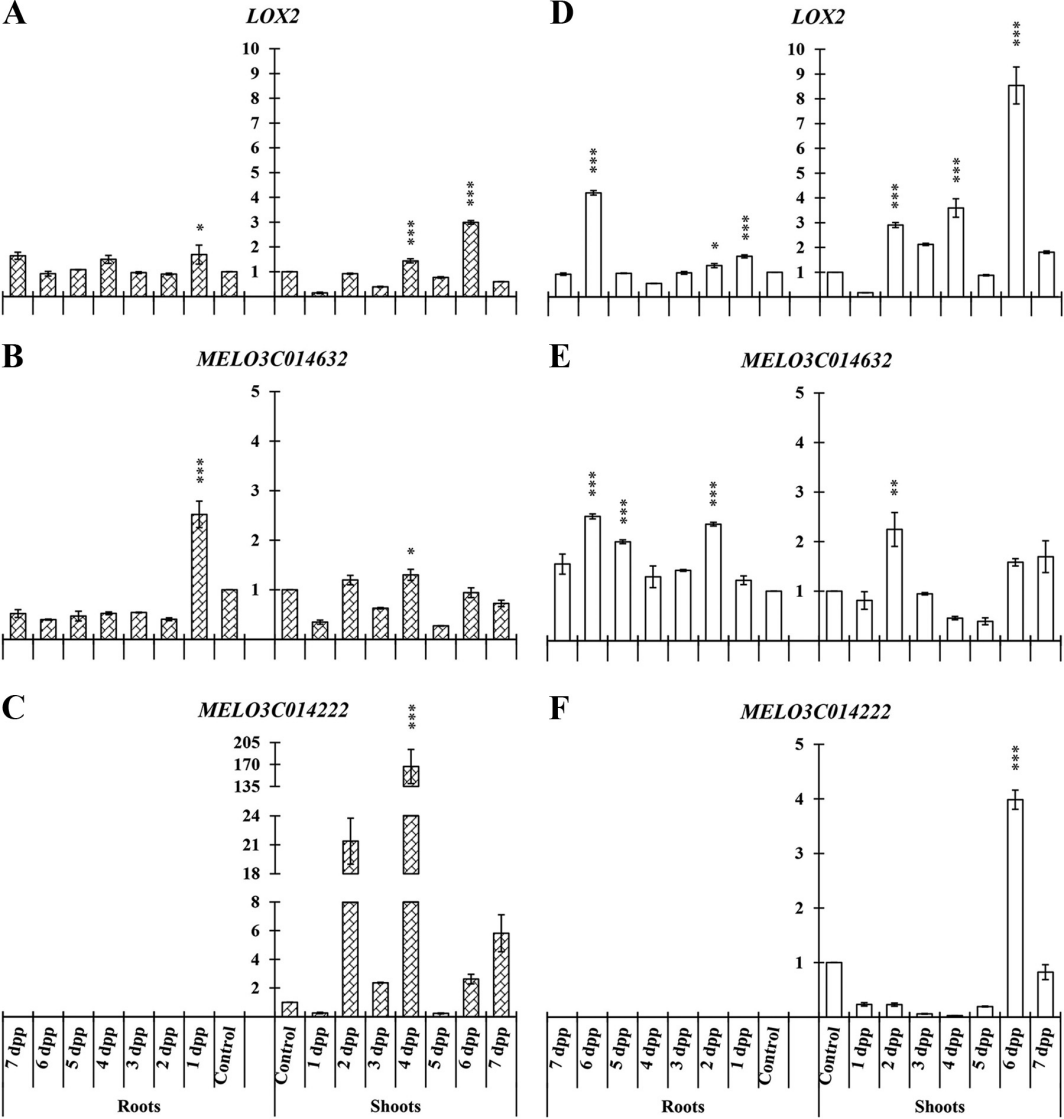
Time course evolution of the relative expression of genes involved in JA (*LOX2* and *MELO3C014632*) and SA (*MELO3C014222*) biosynthesis on roots and shoots of *C. melo*. Plants were primed by root immersion during 30 min in an EAMa 01/58-Su solution with 1 × 10^7^ conidia/mL; plants were maintained in a hydroponic system. Samples were collected during 7 days postpriming for qRT-PCR gene expression study. Patterned bars (left) and white bars (right) represent gene expression of plants grown under Fe-sufficient and Fe-deficient conditions, respectively. Data for *LOX2*, *MELO3C014632*, and *MELO3C014222* expression represent the mean from three independent biological replicates ± SE. The relative expression is based on the expression ratio of a target gene to a reference gene. Bars with *, **, or *** indicate significant differences (*P* < 0.05, *P* < 0.01, or *P* < 0.001, respectively) in relation to their respective control (+Fe40μM or −Fe) according to Dunnett’s test.

A significant increase of *LOX2* and *MELO3C014632* relative expression in roots of primed plants was observed under both nutritional conditions (Fig. 6A, B, D, and E). Likewise, *MELO3COL4222* was not detected in roots.

In melon shoots of primed plants, a significant increment of *LOX2*, *MELO3C014632*, and *MELO3COL4222* could be observed under both nutritional conditions at different times. The relative expression increase of *LOX2* in primed plant shoots under both Fe-sufficient and -deficient conditions reached its maximum at 6 dpp (Fig. 6A and D). Relative expression of *MELO3COL4222* showed a high increase in shoots of primed plants under Fe-sufficient conditions, reaching a maximum relative expression level (165-fold change) at 4 dpp (Fig. 6C).

### Pathogen-related genes.

**FIG 7:**
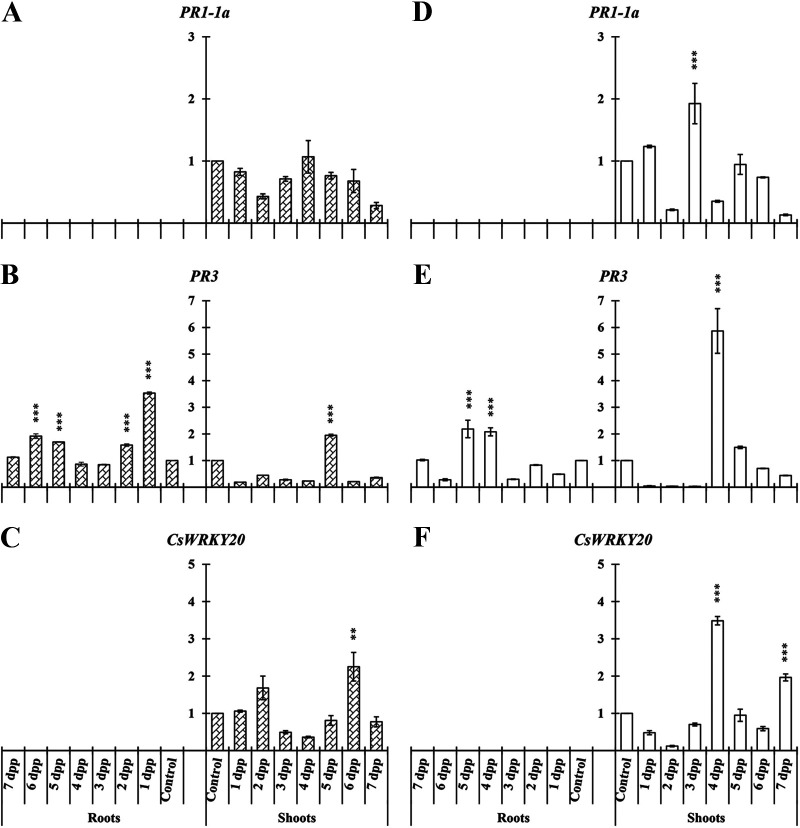
Time course evolution of the relative expression of PR protein genes on *C. sativus* roots and shoots. Plants were primed by root immersion during 30 min in an EAMa 01/58-Su solution with 1 × 10^7^ conidia/mL; plants were maintained in a hydroponic system. Samples were collected during 7 days postpriming for qRT-PCR gene expression study. Patterned bars (left) and white bars (right) represent gene expression of plants grown under Fe-sufficient and Fe-deficient conditions, respectively. Data for *PR1-1a*, *PR3*, and *CsWRKY20* expression represent the mean from three independent biological replicates ± SE. The relative expression is based on the expression ratio of a target gene to a reference gene. Bars with *, **, or *** indicate significant differences (*P* < 0.05, *P* < 0.01, or *P* < 0.001, respectively) in relation to their respective control (+Fe40μM or −Fe) according to Dunnett’s test.

Another important group of genes, PR protein genes, was analyzed in this study. In cucumber we studied *PR1-1a*, *PR3*, and *CsWRKY20.* In roots, *PR3* was the only gene detected under our experimental conditions, showing significant difference under both nutritional conditions (Fig. 7B and E).

In cucumber shoots of primed plants, we found significant differences in relative expression of all three genes studied under both nutritional conditions at different time points, except in *PR1-1a* under Fe-sufficient conditions (Fig. 7A). The relative expression increase of *PR3* and *CsWRKY20* in shoots of primed plants under Fe-deficient conditions reached its maximum at 4 dpp (Fig. 7E and F).

**FIG 8:**
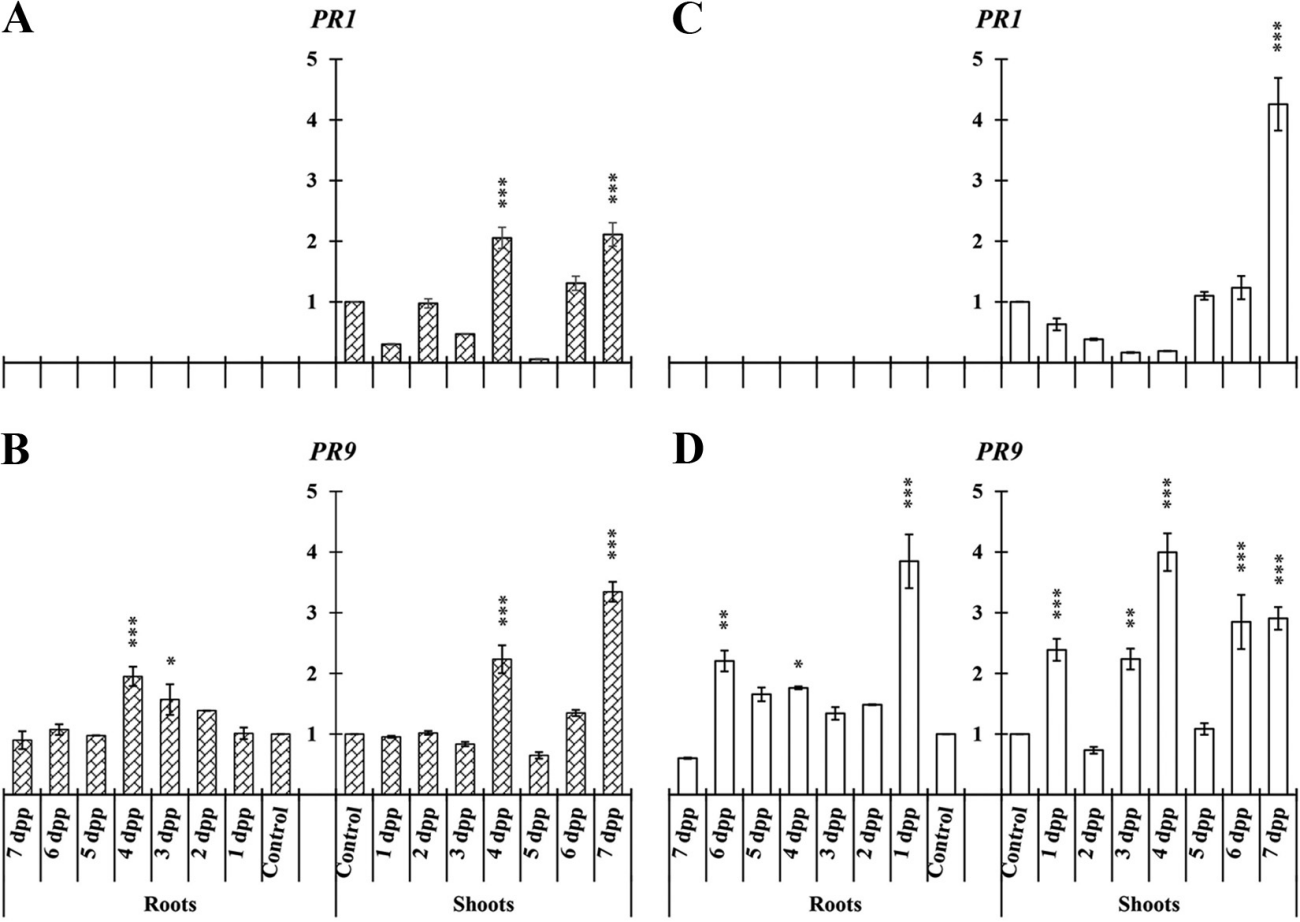
Time course evolution of the relative expression of PR protein genes on *C. melo* in roots and shoots. Plants were primed by root immersion during 30 min in an EAMa 01/58-Su solution with 1 × 10^7^ conidia/mL; plants were maintained in a hydroponic system. Samples were collected during 7 days postpriming for qRT-PCR gene expression study. Patterned bars (left) and white bars (right) represent gene expression of plants grown under Fe-sufficient and Fe-deficient conditions, respectively. Data for *PR1* and *PR9* expression represent the mean from three independent biological replicates ± SE. The relative expression is based on the expression ratio of a target gene to a reference gene. Bars with *, **, or *** indicate significant differences (*P* < 0.05, *P* < 0.01, or *P* < 0.001, respectively) in relation to their respective control (+Fe40μM or −Fe) according to Dunnett’s test.

In the case of melon, the pathogen-related genes studied were *PR1* and *PR9*. *PR1* was detected only in shoots while *PR9* showed significant differences in roots and shoots under both nutritional conditions (Fig. 8).

The induction of *PR9* expression in roots occurred early in primed plants under Fe-deficient conditions while under Fe-sufficient conditions no significant differences were observed until 4 dpp in both roots and shoots (Fig. 8B and D). In shoots, the relative expression of *PR9* increased significantly at most of the time points studied under Fe-deficient conditions (Fig. 8D).

### Relative expression of genes in shoots after second priming.

On the 8th day the second priming of cucumber plants was carried out, and the relative expression of genes involved in ET biosynthesis (*ACO1*, *ACO3,* and *ACS7*) and signaling (*EIN2* and *EIN3*) and JA (*LOX1* and *LOX2*) and SA (*PAL*) biosynthesis was studied only in shoots.

**FIG 9:**
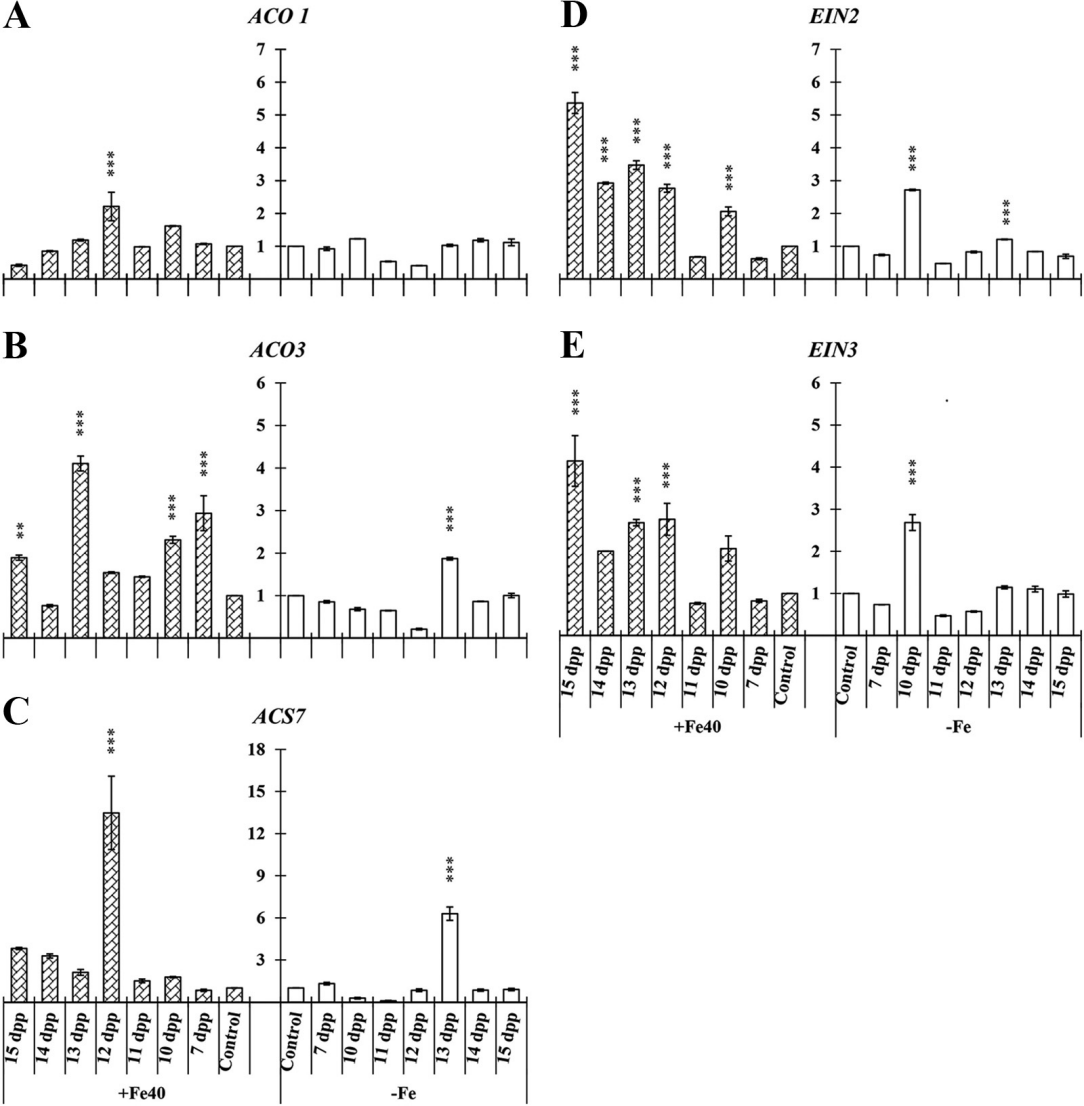
Time course evolution of the relative expression of genes involved in ET biosynthesis (A, B, and C) and ET transduction pathway (D and E) in shoots of *C. sativus*. Plants were primed by root immersion during 30 min in an EAMa 01/58-Su solution with 1 × 10^7^ conidia/mL twice, and the second priming was carried out 8 days after the first one; plants were maintained in a hydroponic system. Samples for the qRT-PCR gene expression study were collected at the 7th day and from 10 to 15 days after the first priming; the second priming was carried out 8 days after the first one. In the same chart, patterned bars and white bars represent gene expression of plants grown under Fe-sufficient and Fe-deficient conditions, respectively. Data for *ACO1*, *ACO3*, *ACS7*, *EIN2*, and *EIN3* expression represent the mean from three independent biological replicates ± SE. The relative expression is based on the expression ratio of a target gene to a reference gene. Bars with *, **, or *** indicate significant differences (*P* < 0.05, *P* < 0.01, or *P* < 0.001, respectively) in relation to their respective control (+Fe40μM or −Fe) according to Dunnett’s test.

*ACO1*, *ACO3*, and *ACS7* significantly increased their relative expression in primed plants under both nutritional conditions (Fig. 9A to C), except in the case of *ACO1*, in which no significant differences were observed under Fe-deficient conditions (Fig. 9A). *EIN2* and *EIN3* relative expression significantly increased in primed plants under both nutritional conditions, reaching the maximum relative expression level under Fe-sufficient conditions at 7 dpp (Fig. 9D and E).

**FIG 10:**
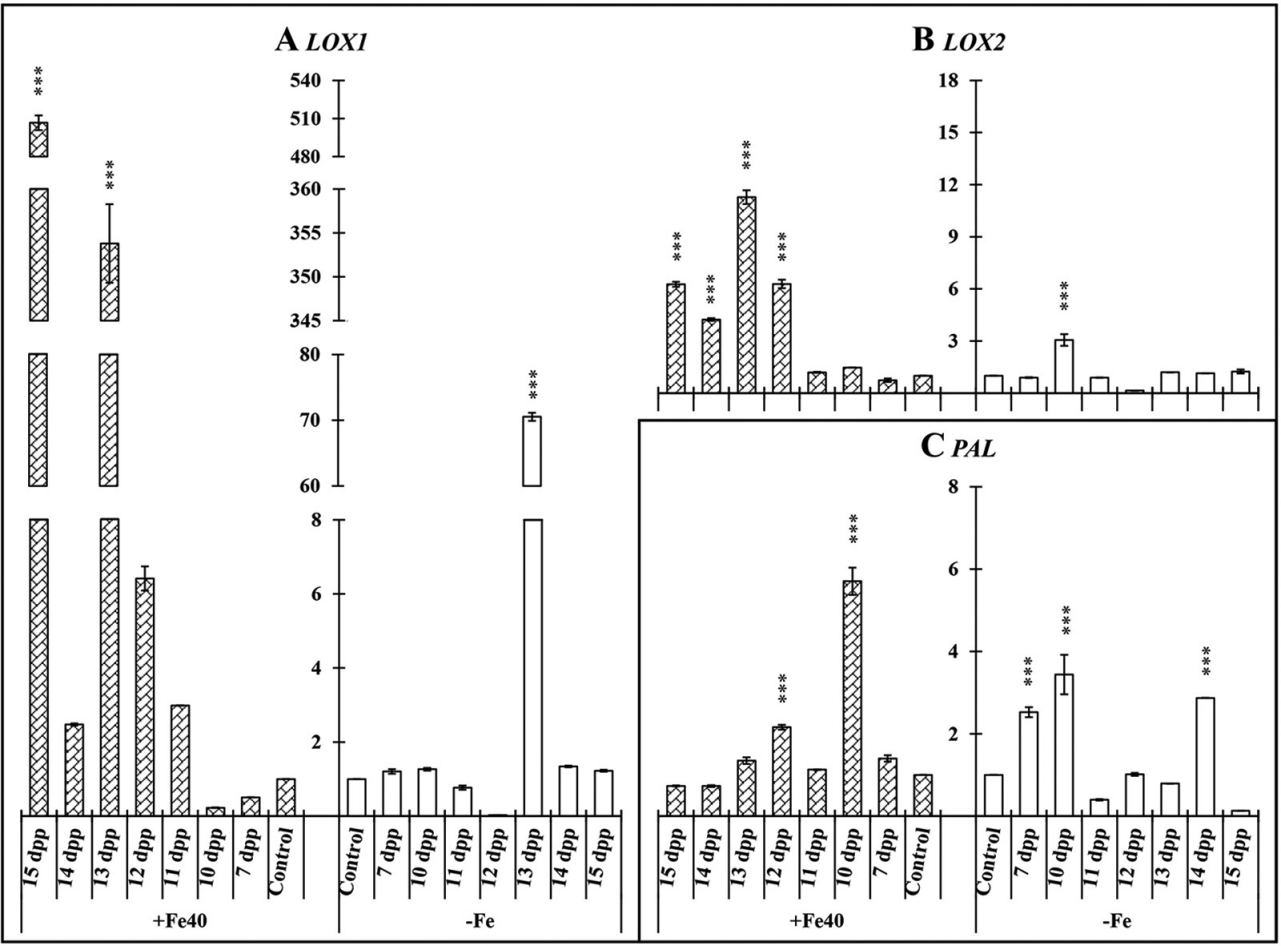
Time course evolution of the relative expression of genes involved in JA (*LOX1* and *LOX2*) and SA (*PAL*) biosynthesis in shoots of *C. sativus*. Plants were primed by root immersion during 30 min in an EAMa 01/58-Su solution with 1 × 10^7^ conidia/mL; plants were maintained in a hydroponic system. Samples for qRT-PCR gene expression study were collected at the 7th day and from 10 to 15 days after the first priming; the second priming was carried out 8 days after the first one. In the same chart, patterned bars and white bars represent gene expression of plants grown under Fe-sufficient and Fe-deficient conditions, respectively. Data for *LOX1*, *LOX2*, and *PAL* expression represent the mean from three independent biological replicates ± SE. The relative expression is based on the expression ratio of a target gene to a reference gene. Bars with *, **, or *** indicate significant differences (*P* < 0.05, *P* < 0.01, or *P* < 0.001, respectively) in relation to their respective control (+Fe40μM or −Fe) according to Dunnett’s test.

*LOX1* relative expression showed a significant increase at 5 dpp under both nutritional conditions, reaching its maximum expression level (506 times) at 7 dpp under Fe-sufficient conditions (Fig. 10A). In the case of *LOX2* and *PAL*, similar results were obtained, and significant differences in relative expression levels were observed in primed plants under both nutritional conditions (Fig. 10B and C).

Finally, the *PAL* gene showed an early relative expression increase under both nutritional conditions, reaching its maximum relative expression level at 2 dpp under Fe-sufficient and -deficient conditions (5.71 and 3.44, respectively) (Fig. 10C).

### Lethal and sublethal effects on *S. littoralis*.

**FIG 11:**
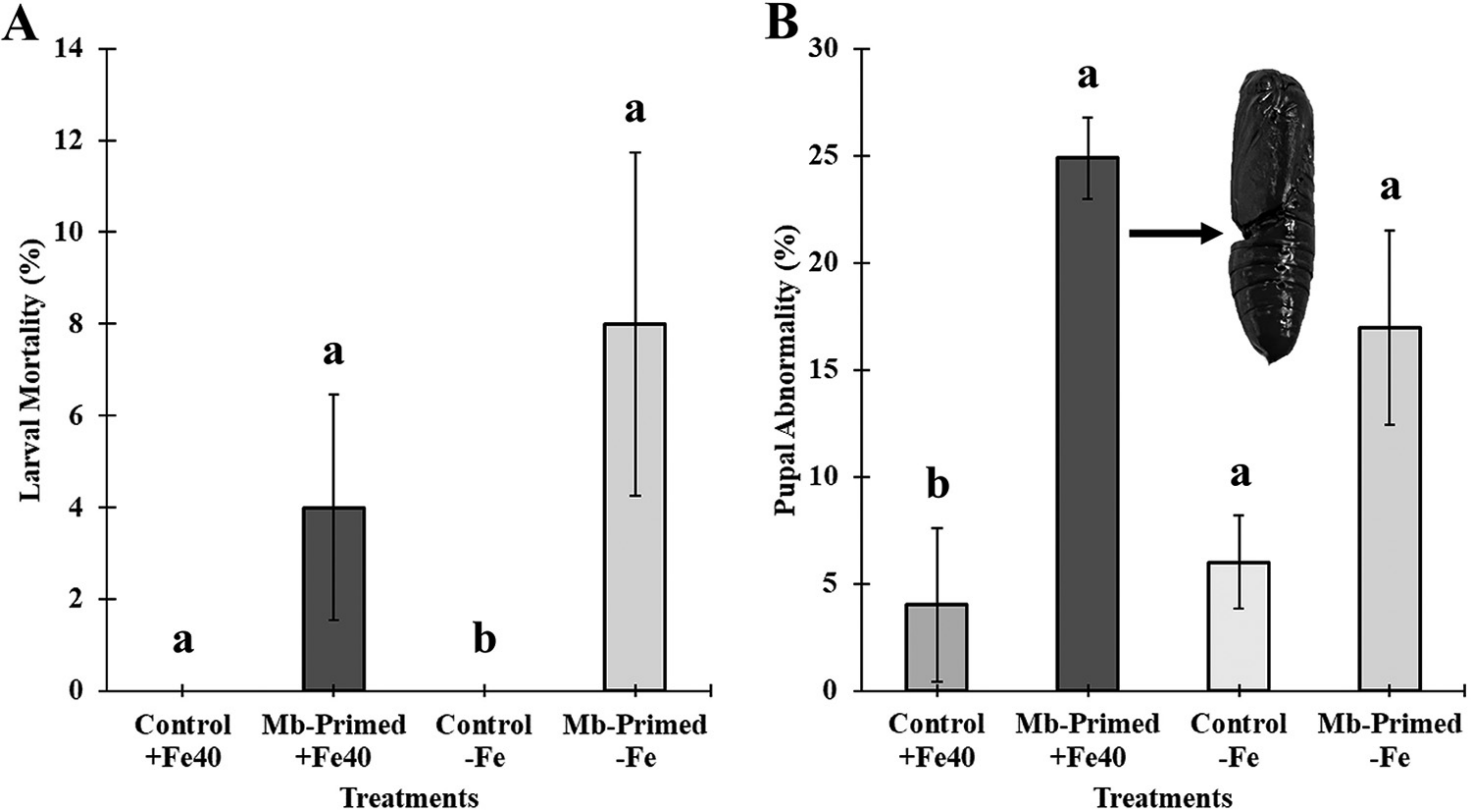
Larval mortality (A) and pupal abnormality (B) of *S. littoralis* insects that were fed during 15 days with fragments of leaves from twice-primed cucumber plants grown under Fe-sufficient and -deficient conditions, the second priming being applied at 8 days after the first one. Four treatments were used, namely, (i) Control +Fe40μM, (ii) Mb-Primed +Fe40μM, (iii) Control −Fe, and (iv) Mb-Primed −Fe. Plants were primed by root immersion during 30 min in an EAMa 01/58-Su solution with 1 × 10^7^ conidia/mL and maintained in a hydroponic system. A letter over the bars denotes a significant difference between each treatment and its respective control; the significance of the treatment was analyzed with the *F* test and Tukey’s multiple comparisons (α < 0.05).

A significant difference was observed in mortality. A 4% mortality rate was recorded in larvae fed with primed plants under Fe-sufficient conditions [χ^2^_(1)_ = 2.73, *P* = 0.0983], while the mortality of larvae fed with primed plants grown under Fe-deficient conditions reached 8% [χ^2^_(1)_ = 5.39, *P* = 0.0202]. In the control treatments (plants without priming) under both nutritional conditions, mortality was 0% (Fig. 11A). Abnormality of pupae presented significant differences only in the case that pupae from larvae fed with primed plants grown under Fe-sufficient conditions reached a 24.88% rate of abnormality [χ^2^_(1)_ = 5.53, *P* = 0.0186], while pupae from larvae fed with primed plants grown under Fe-deficient conditions reached a 16.94% rate of abnormality [χ^2^_(1)_ = 2.51, *P* = 0.1127] versus 4 and 6% in their control, respectively (Fig. 11B).

**FIG 12:**
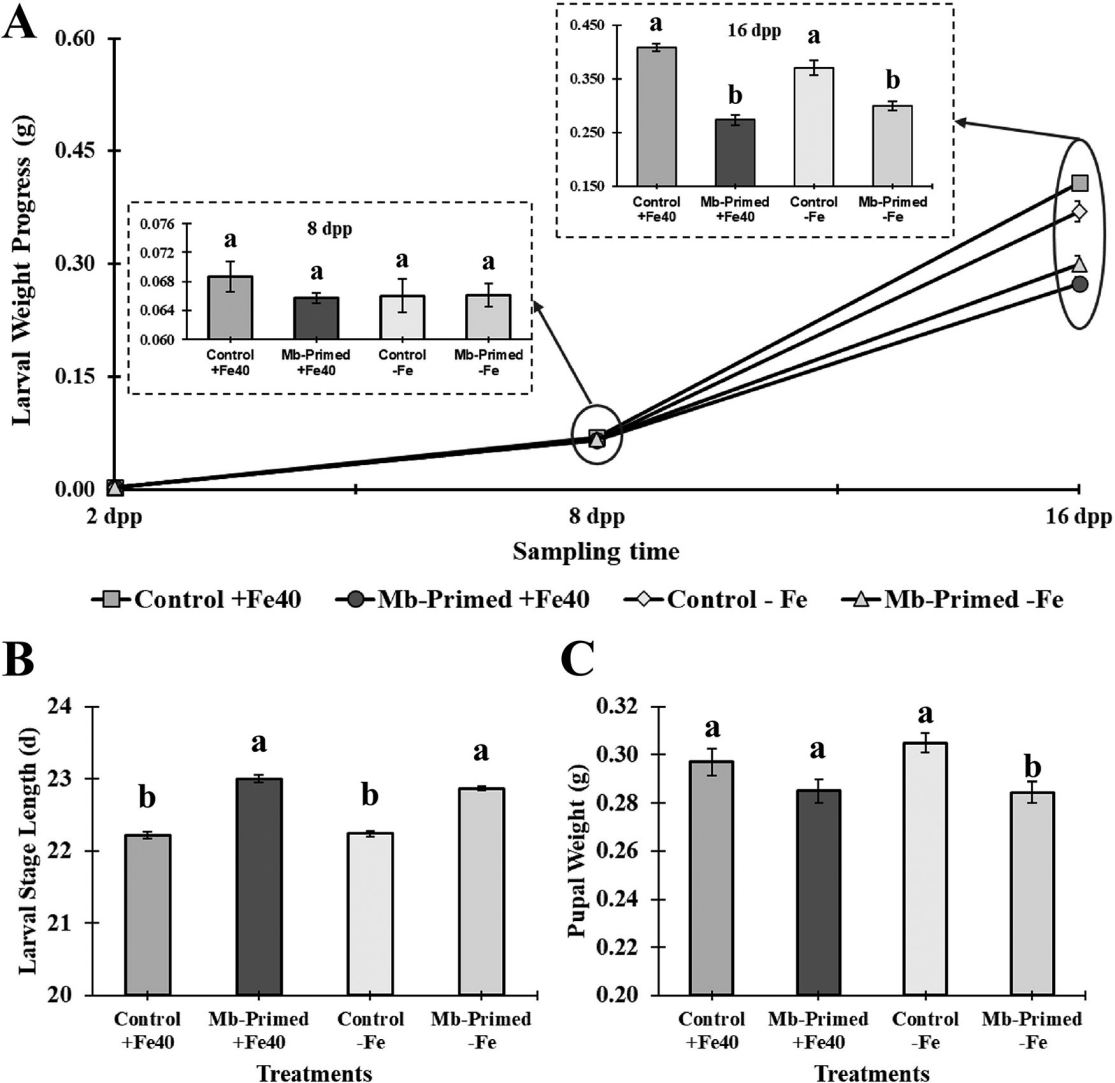
Progress of larval weight (A), larval stage length (B), and weight of pupae (C) of *S. littoralis* larvae that were fed during 15 days with fragments of leaves from twice-primed cucumber plants grown under Fe-deficient and -sufficient conditions; the second priming was applied at 8 days after the first one. Larval stage length represents the time that elapsed from the first day we fed them until the pupal stage was reached. Four treatments were used, namely, (i) Control +Fe40μM, (ii) Mb-Primed +Fe40μM, (iii) Control −Fe, and (iv) Mb-Primed −Fe. Plants were primed by root immersion during 30 min in an EAMa 01/58-Su solution with 1 × 10^7^ conidia/mL and maintained in a hydroponic system. A letter over the bars denotes a significant difference between Mb-Primed plants and their respective control analyzed by completely randomized ANOVA followed by a Tukey test (*P* < 0.05).

The time course evolution of the larval weight showed that there were not significant differences at 8 dpp, while at 16 dpp significant differences among treatments under Fe-sufficient (*F*_1,97_ = 115.31, *P* < 0.001) and Fe-deficient (*F*_1,96_ = 29.76, *P* < 0.001) conditions were recorded, in that larvae fed with primed plants from both nutritional conditions were those that gained less body weight (Fig. 12A). On the other hand, the duration of the larval stage was prolonged by 1 day in those specimens that were fed with primed plants under both Fe-sufficient (*F*_1,96_ = 39.10, *P* < 0.001) and Fe-deficient (*F*_1,94_ = 36.68, *P* < 0.001) conditions (Fig. 12B). Also, pupal weight showed a significant decrease of around 7 to 8% relative to control in the case of Fe-deficient treatment (*F*_1,94_ = 11.63, *P* < 0.001) (Fig. 12C).

### Detection and quantification of *M. brunneum* in shoots.

Despite the effects observed in larvae fed with foliage of primed plants by root immersion, as well as the effects observed in pupae, the presence of *M. brunneum* was not detectable in most of the observation period, although at 6 and 7 dpp, traces of EAMa 01/58-Su DNA could be seen in the collected samples, quantifying minimum concentrations in the range of 0.03 to 0.3 pg from a total of 4 × 10^4^ pg of DNA per PCR. Standard curves generated and other data from quantitative PCR (qPCR) are shown in the supplemental material.

## DISCUSSION

The phenomenon of priming is important for the development of new control methods because priming provides resistance against a broad spectrum of harmful agents significantly affecting growth and fruit or seed production (27, 28, 84, 85). Since it has been shown that priming usually involves epigenetic changes, a transgenerational priming phenomenon can occur (27, 86, 87), as has been shown in several works with natural and chemical compounds (86), with microorganisms such as Pseudomonas syringae (88–90), or by herbivore attack (85, 88). Recent works have demonstrated that evolutionary relatives of Metarhizium, such as Trichoderma atroviride, can transmit the priming and the plant growth promotion effect to the next generation (36, 91); however, the inherited priming phenomenon does not always take place, since experimental conditions can be decisive (92). Furthermore, the priming and subsequent induction of resistance and Fe acquisition-related genes are usually interconnected by common regulators such as ET, JA, and NO, and it has even been recently shown that the inoculation of Arabidopsis thaliana plants with Botrytis cinerea activates Fe deficiency and resistance responses to *B. cinerea* through the induction of ET biosynthesis genes *SAM1* and *SAM2* (52, 93).

In the case of EPF, priming could be a very interesting strategy as it is an added value for the fungal efficacy when used for pest control. Usually, microorganism inducers of ISR also promote plant growth and development (77) and favor Fe acquisition (18, 78–83). In the present work, we show the ability of the EPF *M. brunneum* to trigger both SA- and JA/ET-dependent priming in cucumber and melon seedlings with lethal and sublethal effects on *S. littoralis* fed on primed plants. In other works it is demonstrated that at short times, *Trichoderma* induces SA-dependent defenses and then later activates JA/ET-dependent defenses (94, 95), similarly to what occurred in our study with *M. brunneum*. In the case of *Trichoderma* it is accepted that the plant can modulate *Trichoderma*-activated priming depending on the pathogen cycle, as is the case with root knot nematodes (RKN) (91, 96), and four timing stages can be identified (38). In addition, the fluctuating defense response is also effective against abiotic stresses and is more evident when the stress is present (97). Other beneficial fungi, such as EPF, can be expected to exert similar positive effects to a greater or lesser extent.

In general, gene expression levels obtained in cucumber and melon were similar, with a clear induction of all SA, JA, ET, and PR protein genes in shoots of both plant species studied at different times after root priming. The results obtained show the cross talk among ISR and the nutritional status of plants since in general we observed higher relative expression levels in shoots of primed plants under Fe-deficient conditions over the 7 days after the first priming. However, after the second priming, the tendency changed and higher relative expression levels of JA, SA, and ET-related genes were observed in shoots of primed plants under Fe-sufficient conditions. Our results demonstrated induction of the expression of ET biosynthesis genes (*ACO1*, *ACO3*, and *ACS7* from cucumber and *ACO1*, *ACO3*, *ACO5*, and *ACS7* in melon) in shoots and roots of primed plants under both nutritional conditions at different times over the 15 monitored days. Besides ET biosynthesis, relative expressions of ET signaling pathway genes, *EIN2* and *EIN3*, two key proteins in the ET signaling pathway, and *MELO3CO19787*, which encodes an ERF transcription factor, were significantly induced in shoots and roots under both nutritional conditions. These results would indicate that *M. brunneum* priming affects not only ET biosynthesis but ET signaling, therefore making plants more sensitive to this hormone. These results are in concordance with those of Aparicio et al. (78), who have studied several genes related to ET biosynthesis (*ACO1* and *ACO3*) and signaling (*EIN2* and *EIN3*) in cucumber roots in Fe-sufficient and -deficient plants inoculated with the nonpathogenic strain Fusarium oxysporum FO12 over 4 days and found a significant induction of the genes studied in inoculated plants at different times independently of the nutritional status.

On the other hand, we studied several JA and SA-biosynthesis-related genes in cucumber (*LOX1*, *LOX2*, and *PAL*) and melon plants (*LOX2* and *MELO3CO14632*) with an important and significant increase in shoots of primed plants. However, the relative expression of JA and SA in roots was also enhanced by *M. brunneum* in comparison with their respective controls. For some genes like *MELO3CO14222*, which encodes phenylalanine ammonia lyase, an enzyme involved in SA biosynthesis, the expression was detected only in shoots with a high relative expression level reaching 164-fold change at 4 dpp under Fe-sufficient conditions. Similar results were obtained with PR protein-encoding genes *PR3*, *PR1-1a*, and *CsWRKY20* in cucumber and *PR1* and *PR9* in melon plants; their relative expression levels were enhanced in roots and shoots of primed plants under both nutritional conditions. It is worth noting that the second priming led to an additional enhancement of expression level, namely, the case of *LOX1*, whose relative expression values reached more than a 500-fold change at 15 dpp. These results suggest that the optimization of application times would play a very important role in the resistance induction. In general, little is known about the role of EPF as ISR inducers. Some works have revealed that endophytism by Beauveria bassiana, *M. brunneum*, and Metarhizium robertsii leads to an increase in the relative expression of ET (*ERF-1*, *ACS1*, *WRKY51*), JA (*LOX1*, *LOXF*, *AOS*, *AOC*, *OPR7*, *MPI*, *JAZ1-5A*) and SA (*PR1*, *PR1-1-like*, *PR2*, *PR4, PR5*, *BGL*, *PAL*, *PBS1*) pathway-associated genes in grapevine, faba beans, maize, tomato, and wheat (26, 42, 43, 98), whereas the possible impact of such induction on insect survival and fitness remains unknown.

Our study shows lethal and sublethal effects on *S. littoralis* fed with shoots of primed cucumber plants. Even if mortality rates were not too high (up to 8%), significant sublethal effects were recorded with decreased larval and pupal weight, increased larval development time, and abnormality of pupae. The efficacy of the EAMa 01/58-Su strain of *M. brunneum* has been demonstrated against noctuid larvae with high mortality values when directly applied to the insect larvae (up to 80%) (5), when larvae were fed with treated plants (up to 50%) (7), and when larvae were fed with or endophytically colonized plants (up to 20%) (2). However, similar to our study, no fungal outgrowth was recorded in any of the dead larvae. In this sense, larval mortality could be explained by the capacity of this strain to produce destruxin toxins (99, 100) or by the ISR-SAR induction as shown in the present work in which larvae were fed with leaves of noncolonized primed plants that showed high relative expression levels of several genes related to ET biosynthesis (*ACO1*, *ACO3*, and *ACS7*) and signaling (*EIN2* and *EIN3*) and JA and SA biosynthesis (*LOX1*, *LOX2*, and *PAL*). In this regard our work shows that the lethal and sublethal effects recorded were a direct consequence of *M. brunneum* priming. Related studies indicated that the effects on insect pests are outputs of endophytic colonization and the subsequent enhancement of ISR induction (42, 101–103). Likewise, other studies reported upregulation of ET, JA, SA, and PR-related genes as endogenous responses of resistant genotypes against phytopathogens like *Phytophthora capsici* and *P. melonis* (48, 49) or as a result of the inoculation/interaction with other microorganisms like bacteria (32) or mycorrhizal fungi (50). Recently, Di Lelio et al. (40) showed very similar lethal and sublethal effects on *S. littoralis* larvae fed on tomato plants treated by seed coating with Trichoderma afroharzianum. However, these effects were attributed to gut dysbiosis as a result of plant colonization which led to resistance enhancement. In our study, we showed that the ISR-SAR induction is not necessarily related to endophytic colonization and may cause important effects on insect pest fitness.

### Conclusions.

Our results evidence the role of the *M. brunneum* EAMa 01/58-Su strain as an ISR-SAR inducer, by triggering both SA- and JA/ET-dependent priming, and the benefits of this resistance activation for *S. littoralis* management. Also, the cross talk between the ISR-SAR induction, insect pest control, and the Fe nutritional status of the plant is highlighted. This study contributes to the knowledge of new functions of EPF that could be integrated as innovative IPM strategies.

## MATERIALS AND METHODS

### Biological material.

Two species of Cucurbitaceae (Cucumis melo L. var. Futuro and Cucumis sativus L. var. Ashley; Semillas Fitó, S.A., Barcelona, Spain) and *S. littoralis* (Lepidoptera: Noctuidae) were used to study the effects of priming with an entomopathogenic fungus on the responses and expression of both induced and acquired systemic resistance.

### Growth conditions.

Plants were grown under controlled conditions as described by García et al. (104). Briefly, seeds of both species were sterilized with 1% sodium hypochlorite for 5 min, with constant stirring, then washed twice with sterilized water and placed on absorbent paper moistened with 5 mM CaCl_2_, covered with the same paper, and held at 25°C in the dark over 3 days for germination. Then, when the plants sufficiently elongated their stems, they were transferred to a hydroponic system culture that consisted of a thin polyurethane raft with holes on which plants inserted in plastic lids were held floating on the aerated nutrient solution. Plants grew in a growth chamber at 22°C (day)/20°C (night) temperatures, with relative humidity (RH) between 50 and 70%, and a 14-h photoperiod at a photosynthetic irradiance of 300 μmol m^−2^ s^−1^ provided by white fluorescent light (10,000 lx).

The nutrient solution used was R&M (105), whose composition is the following: macronutrients, 2 mM Ca(NO_3_)_2_, 0.75 mM K_2_SO_4_, 0.65 mM MgSO_4_, 0.5 mM KH_2_PO_4_, and micronutrients, 50 μM KCl, 10 μM H_3_BO_3_, 1 μM MnSO_4_, 0.5 μM CuSO_4_, 0.5 μM ZnSO_4_, 0.05 μM (NH_4_)6Mo_7_O_24_, and 10 μM Fe-EDDHA [ethylenediaminedi(*O*-hydroxyphenylacetic) acid].

After 10 days and 13 days of growth, for cucumber and melon, respectively, plants were separated into four groups that subsequently constituted the 4 treatments, as described below.

The specimens of *S. littoralis* used in this work came from a colony established at the insectarium of the Agricultural and Forestry Entomology Laboratory of the University of Córdoba; the growth chamber was maintained under the following conditions: 26 ± 2°C, 70% ± 5% relative humidity (RH), and a photoperiod of 16:8 h (light [L]/dark [D] ratio) (2, 106).

### Fungal strain and inoculum preparation.

A Metarhizium brunneum (EAMa 01/58-Su) strain from the culture collection of the Agronomy Department, University of Córdoba (Spain), was used in all experiments (Spanish Type Culture Collection accession number 20764). Detailed information about the fungal strain can be found in the work of García-Espinoza et al. (18). Transient and temporary endophytic colonization of melon plants by this strain has been previously demonstrated in foliar application (5, 6), and the positive effects on growth promotion and response to Fe deficiency of *M. brunneum* have been described previously (107–109) in several cultivated species. Recently, we unraveled the direct and indirect mechanisms used by this strain for Fe acquisition by cucurbits (18).

To provide an inoculum for experiments, the strain was subcultured from stored slant cultures on potato dextrose agar (PDA) in petri dishes and grown for 15 days at 25°C in darkness. Then, inoculum preparation was carried out by scraping the conidia from the petri plates into a sterile solution of 0.1% Tween 80, followed by sonication for 5 min to homogenize the inoculum and filtration through several layers of cheesecloth to remove any mycelia. A hemocytometer (Malassez chamber; Blau Brand, Wertheim, Germany) was used to estimate conidial concentration, which was finally adjusted to 1 × 10^7^ conidia/mL by adding a sterile solution of distilled water with 0.1% Tween 80.

### Root priming.

Melon and cucumber plants with two true leaves were selected and placed in trays with 2.5 L of fungal inoculum suspension, previously adjusted to 1 × 10^7^ conidia/mL. Control plants (nonprimed) were placed in trays with 2.5 L of 0.1% Tween 80. All plants were maintained in continuous agitation for 30 min. After that, EAMa 01/58-Su-primed plants, here called Mb-Primed, and nonprimed plants were transferred to two different nutritional conditions, Fe sufficient (+Fe40μM) and deficient (−Fe), so that finally four treatments were used: Control +Fe40μM (nonprimed), Mb-Primed +Fe40μM, Control −Fe (nonprimed), and Mb-Primed −Fe.

### Relative expression of defense mechanism-related genes and effects on *S. littoralis* fitness.

**FIG 13:**
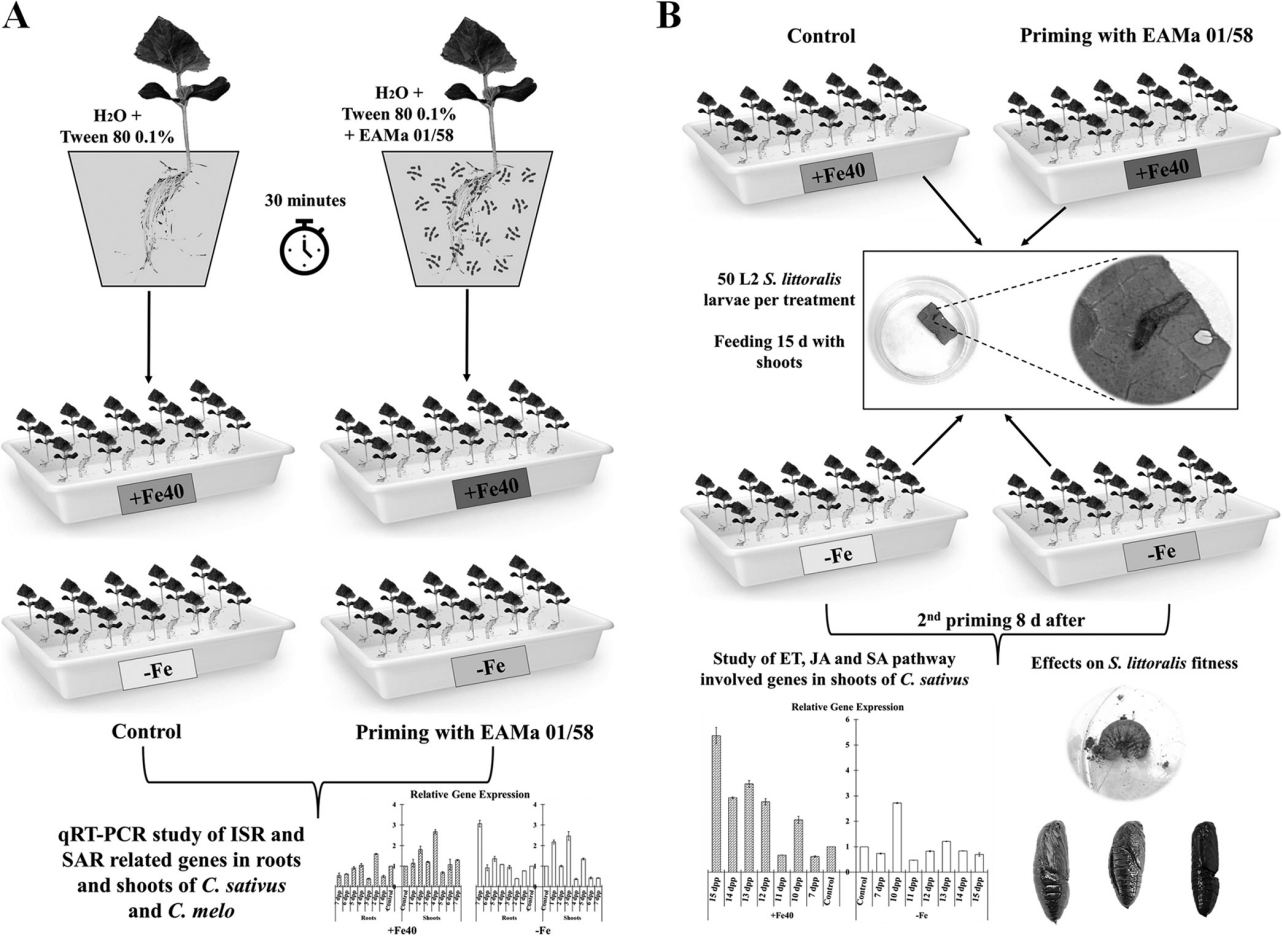
Scheme of treatments and priming carried out in assays. In the first group of assays, including *C. sativus* and *C. melo*, plants were primed by root immersion during 30 min in an EAMa 01/58-Su suspension with 1 × 10^7^ conidia/mL; plants were maintained in a hydroponic system. Four treatments were used, namely, (i) Control +Fe40μM (nonprimed), (ii) Mb-Primed +Fe40μM, (iii) Control −Fe (nonprimed), and (iv) Mb-Primed −Fe. Samples were collected during 7 days postpriming for qRT-PCR study of genes related to ISR and SAR (A). In order to study the effects of priming on fitness of *S. littoralis*, 50 L2 larvae per treatment were fed with shoots of control and primed *C. sativus* plants during 15 days; priming was carried out twice, and the second one was carried out at 8 days after the first one; for that, the roots were immersed for 30 min in an EAMa 01/58-Su suspension with 1 × 10^7^ conidia/mL (B).

In a first series of experiments, the relative expression levels of 18 ISR- and SAR-related genes were studied over the 7 days postpriming (dpp) without insect pest presence. For that, samples of roots and shoots, separately, were collected daily from 1 to 7 dpp, frozen immediately with liquid nitrogen, and subsequently stored at −80°C. A total of 42 plants were used for each treatment and plant species (6 plants per day and treatment for each plant species). The whole assay with both species of *Cucumis* was repeated twice (Fig. 13A).

Based on the gene expression results obtained, *C. sativus* was chosen for this part of the study. A group of cucumber plants were used to study the impact of root priming by the fungus on *S. littoralis* fitness. For that, plant roots were primed as previously described, and at 2 dpp, 50 larvae of *S. littoralis* (L2) were introduced and confined in methacrylate boxes to observe larval development. A second priming was applied to the roots 8 days after the first one. Larvae were fed daily with fragments of leaves of plants from their respective treatment over 15 days. After these 15 days, the larvae were fed with an artificial diet until they reached the pupal stage. Larval mortality and development were monitored daily. The larval stage length, pupal abnormality, and pupal weight were recorded; the larvae were weighed at the beginning of the study and at 8 and 16 dpp. The assay was set up into four treatments, as previously explained, with 5 replicates (10 larvae per replicate). The relative expression of genes was studied only in shoots after the second priming (Fig. 13B).

### RNA isolation, cDNA synthesis, and reverse transcription-quantitative PCR (qRT-PCR) analysis.

Real-time PCR analysis was carried out as previously described by García et al. (104). Briefly, roots and leaves were ground to a fine powder with a mortar and pestle in liquid nitrogen. Total RNA was extracted using the Tri reagent solution (Molecular Research Center, Inc., Cincinnati, OH, USA) according to the manufacturer’s instructions. cDNA synthesis was performed by using the iScript cDNA synthesis kit (Bio-Rad Laboratories, Inc, Hercules, CA, USA) from 3 μg of DNase-treated RNA as the template. As an internal control, 18S cDNA was amplified using the QuantumRNA Universal 18S Standards primer set (Ambion, Austin, TX, USA); the thermal cycler program was one initial cycle of 94°C for 5 min, followed by cycles of 94°C for 45 s, 55°C for 45 s, and 72°C for 1 min, with 27 to 30 cycles, all followed by a final 72°C elongation cycle of 7 min (110–113).

The study of gene expression by qRT-PCR was performed in a qRT-PCR Bio-Rad CFX Connect thermal cycler and the following amplification profile: initial denaturation and polymerase activation (95°C for 3 min), amplification and quantification repeated 40 times (94°C for 10 s, 57°C for 15 s, and 72°C for 30 s), and a final melting curve stage of 65°C to 95°C with an increase of 0.5°C for 5 s to ensure the absence of primer dimer or nonspecific amplification products (104). PCR mixtures were set up with 2 μL of cDNA in 23 μL of SYBR green Bio-Rad PCR master mix, following the manufacturer’s instructions. Standard dilution curves were performed for each primer pair to confirm appropriate efficiency of amplification (*E* = 100% ± 10%). Relative expression levels of ethylene- and jasmonic and salicylic acid-related genes as well as genes that encode PR proteins were studied in roots and shoots of both species, *C. sativus* and *C. melo*. Constitutively expressed *ACTIN* and *CYCLO* genes were used as reference genes to normalize qRT-PCR results. The relative expression levels were calculated from the threshold cycle (*C_T_*) values and the primer efficiencies by the Pfaffl method (114). Each PCR analysis was conducted on three biological replicates, and each PCR was repeated twice.

The primers used in this study are listed in Table 1. Oligonucleotides used to amplify *ACO5*, *ACS2*, *ACS7*, *LOX2* (for cucumber), and *PAL* were designed by using Primer-Design software on the NCBI site (115).

**TABLE 1:** Gene names, accession numbers, and forward and reverse primer sequences studied on *C. melo* and *C. sativus* root and shoot samples

Hormone and gene	Gene name/function	Accession no.	Reference	Sequence^*a*^	Species
Ethylene					
*ACO1*	1-Aminocyclopropane-1-carboxylic acid oxidase 1	FN544066	78	F: TTTGGTGGCGGAGGAGAAAA R: ATGGCTTCAAACCTCGGCTC	*C. melo/C. sativus*
*ACO3*	1-Aminocyclopropane-1-carboxylic acid oxidase 2	AF033583	78	F: ACTCAAAACAGTGGAACTGGA R: GGGGTACACTTCCTTCTTCTCC	*C. melo/C. sativus*
*ACO5*	1-Aminocyclopropane-1-carboxylic acid oxidase 5	XM_008445975.2		F: AGCAAACCAGGAAGTGGAAGA R: GCTCCTCACATTGCTCTGAC	*C. melo*
*ACS7*	1-aminocyclopropane-1-carboxylic acid synthase 7	NM_001328455.1		F: CTCGCCGGATGTCTAGCTTT R: AGCCTGTCCCGGTTCATTTT	*C. melo/C. sativus*
*EIN2*	Ethylene-insensitive protein 2	KF245636	78	F: TGCCGACAAGGTTAAATGGG R: TGCTGCTGCACAATAGAAGA	*C. melo/C. sativus*
*EIN3*	Ethylene-insensitive protein 3	KF245636	78	F: GCTTTCTGGGGTTGCGATTT R: CCGAACAGTCTCCCAAAGCA	*C. melo/C. sativus*
*MELO3C019787*	AP2-like ethylene-responsive transcription factor		49	F: CTTCGTTTTCCTATCTTCCAATCC R: CATCAACAAAGTCAAGTAGCCCTC	*C. melo*
Jasmonic acid					
*LOX1*	Lipoxygenase 1	XM_004139124.1	48	F: TCTTTGCTTCAGGGTATCAC	*C. sativus*
				R: GCAAATTCTTCATCACTACTCC	
*LOX2 (Cs)*	Lipoxygenase 2	NM_001305766.1		F: GCACTTTGAGCATGTGGTTG	*C. sativus*
				R: AAGCTACTCTAAAGCACTCTTTTCT	
*LOX2 (Cm)*	Lipoxygenase 2	GQ386815	32	F: GCGTAAGGAATGGGATAGAATATATGA	*C. melo*
				R: CGACGAGGATAAGGGAATTGG	
*MELO3C014632*	Linoleate 13S-lipoxygenase 2-1		49	F: AACGCCTTTCGCTGCTT	*C. melo*
				R: TGTAGGACTCTGGTGGTGGA	
Salicylic acid					
*PAL*	Phenylalanine ammonia lyase	NM_001308910.1		F: TCACTCCGCAACACGAGCA	*C. sativus*
				R: GGAGTGACGTTGTGGTTCAAG	
*MELO3C014222*	Phenylalanine ammonia lyase		49	F: ATTTTGTCGGGCATCTTTG	*C. melo*
				R: GCGATCTTGTTTTGGCTTCT	
PR proteins					
*PR3*	Pathogenesis-related protein 3	NM_001308904.1	48	F: CACTGCAACCCTGACAACAACG	*C. sativus*
				R: AAGTGGCCTGGAATCCGACTG	
*PR1-1a*	Pathogenesis-related protein 1-1a	AF475286.1	48	F: CTCAAGACTTCGTCGGTGTCCA	*C. sativus*
				R: CGCCAGAGTTCACTAGCCTAC	
*CsWRKY20*	WRKY transcription factor of PR protein	XM_011653112.1	48	F: GAAATAACGTACAGAGGGAAGC R: CAGGTGCTGTTTGTTGGTTATG	*C. sativus*
*PR1*	Pathogenesis-related protein 1	EU556704	32	F: GAGTGGGACAGAATAGTAGCAGGTT	*C. melo*
				R: GTGCACTAGCCTACAGTCGTTGA	
*PR9*	Pathogenesis-related protein 9	AY373372	32	F: GCATCTCGATCGTCCAAATGT	*C. melo*
				R: TTGGGCTCAATACCGTGGAT	
Constitutive genes					
*Actina*	Actina	XM_004136807	78	F: AACCCAAAGGCAAACAGGGA	*C. melo/C. sativus*
				R: TCCGACCACTGGCATAGAGA	
*Cyclo*	Cyclophilin	NM_001280769	78	F: ATTTCCTATTTGCGTGTGTTGTT	*C. melo/C. sativus*
				R: GTAGCATAAACCATGACCCATAATA	

a F, forward; R, reverse.

### Detection and quantification of *M. brunneum* by quantitative PCR. (i) DNA isolation.

For each treatment, namely, Control +Fe40μM (nonprimed), Mb-Primed +Fe40μM, Control −Fe (nonprimed), and Mb-Primed −Fe, samples were collected from remains after feeding and stored at −20°C, from 2 to 7 dpp. After each sampling, vegetal material was surface sterilized with 1% sodium hypochlorite for 2 min, rinsed twice in sterile deionized water for 2 min each, and dried on sterile filter paper (2, 116).

Plant material was ground to a fine powder with a mortar and pestle in liquid nitrogen. Total DNA was isolated using the HigherPurity plant DNA purification kit (Canvax Biotech S.L., Córdoba, Spain) according to the manufacturer’s instructions and resuspended in 100 μL of elution buffer. The concentration and quality of DNA were assessed by determination of absorbance at 260 nm and 280 nm in a NanoDrop 2000 (Thermo Fisher Scientific Inc.). The final concentration was homogenized to 30 ng/μL.

### (ii) Quantitative PCR.

The specific primer of the *nrr* gene (forward, TCA GGC GAT CTC GTG GTA AG; reverse, GGG GTG TAC TTG AGG AAT GGG) for qPCR was used (117). Real-time PCR was performed in a qRT-PCR Bio-Rad CFX Connect thermal cycler, and the appliance was set to the following amplification profile: initial denaturation and polymerase activation (95°C for 3 min), amplification and quantification repeated 40 times (94°C for 10 s, 65°C for 15 s, and 72°C for 30 s), and a final melting curve stage of 65°C to 95°C with an increase of 0.5°C for 5 s to ensure the absence of primer dimer or nonspecific amplification products. PCR mixtures were set up with 1.3 μL of the template (40 ng total) in 18.7 μL of SYBR green Bio-Rad PCR master mix, following the manufacturer’s instructions.

Absolute quantification was carried out according to the work of Bell et al. (118) and Barelli et al. (117). A gradient of 1:4 from 40 ng to 0.61 pg of fungal and plant genomic DNA was used to set up standard curves; absolute quantification was determined by comparing threshold cycle numbers against the standard curve previously generated (117, 118).

### Statistical analysis.

All assays were carried out twice, and representative results of both species studied are presented. The values of qRT-PCR represent the mean ± standard error (SE) from three independent technical replicates. Results of relative expression were analyzed using one-way analysis of variance (ANOVA) followed by Dunnett’s test. * (*P* < 0.05), ** (*P* < 0.01), or *** (*P* < 0.001) over the bars in the figures indicates significant differences in relation to the control treatment (GraphPad Prism 9.4.0; GraphPad Software, LLC, San Diego, CA, USA).

Data for mortality and abnormality of pupae, expressed as percentages, were analyzed using a generalized linear mixed model with binomial distribution and logit link function. Significance of the treatment was analyzed with the *F* test and Tukey’s multiple comparisons (α < 0.05) (JMP 8.0; SAS Institute Inc.). Data for weight of pupae and larval stage duration were analyzed using analysis of variance (ANOVA) followed by a Tukey multiple-range test; different letters over the bars in the figures indicate significant differences (*P* < 0.05) among treatments (Statistix 9.0; Analytical Software, Tallahassee, FL, USA).

### Data availability.

The data of the present study are in the possession of the authors and are available for consultation under the respective request; for any additional information, please contact the corresponding author.
