# Supplementary material for: Entomopathogenic Fungus-Related Priming Defense Mechanisms in Cucurbits Impact Spodoptera littoralis (Boisduval) Fitness
Source: Appl Environ Microbiol. 2023 Jul 13;89(8):e00940-23. doi: 10.1128/aem.00940-23 (PMC10467339; doi:10.1128/aem.00940-23)
Supplement: Supplemental file 1 — Fig. S1 and S2. Download aem.00940-23-s0001.docx, DOCX file, 0.6 MB [file aem.00940-23-s0001.docx]

Entomopathogenic fungus-related priming defense mechanisms in cucurbits impacts on *Spodoptera littoralis* (Boisduval) fitness

**García-Espinoza, F.^1,2, †^, García, M.J.^1, †^, Quesada-Moraga, E.^1^ and Yousef-Yousef, M.^1,^ ***

^1^Departamento de Agronomía (DAUCO) María de Maeztu Unit of Excellence 2021–2023), Campus de Rabanales, Universidad de Córdoba, Edif. C4, 14071 Córdoba, España.

^2^Departamento de Parasitología. Universidad Autónoma Agraria Antonio Narro – Unidad Laguna. Periférico Raúl López Sánchez S/N, 27054 Torreón, Coahuila, México.

† García-Espinoza and García contributed equally to this work. Author order was determined in order of increasing seniority.

*** Correspondence:**Meelad Yousef-Yousef
z12yonam@uco.es

ORCID: FGE 0000-0002-1356-1048; MJG 0000-0002-2863-7922; EQM 0000-0003-4021-3900; MYY 0000-0002-1530-4483


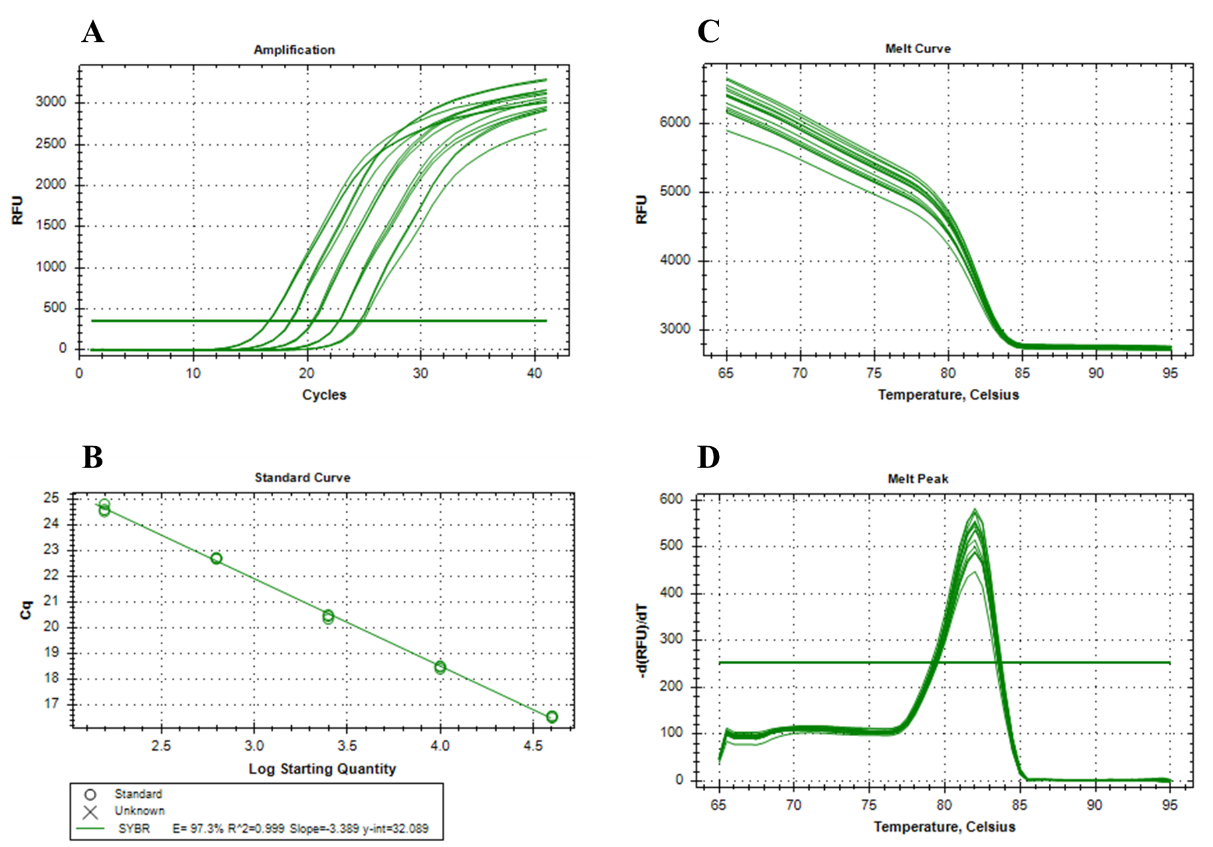


**FIG S1.** Output data from qPCR for molecular detection and quantification of *Metarhizium brunneum*. Quantification at gradual increment of number of cycles along the gradient of serial dilution (1:4 fold) (A). Figure B shows standard curve generated from serial dilutions of fungal DNA+DNA of *C. sativus* var. Ashley. Efficiency of primer was 97.3% while R^2^ obtained was equal to 0.999. In Figures C and D show melting curve and melting peak, both shows the high specificity of *nrr* gene primer to identify *M. brunneum* EAMa 01/58-Su strain.


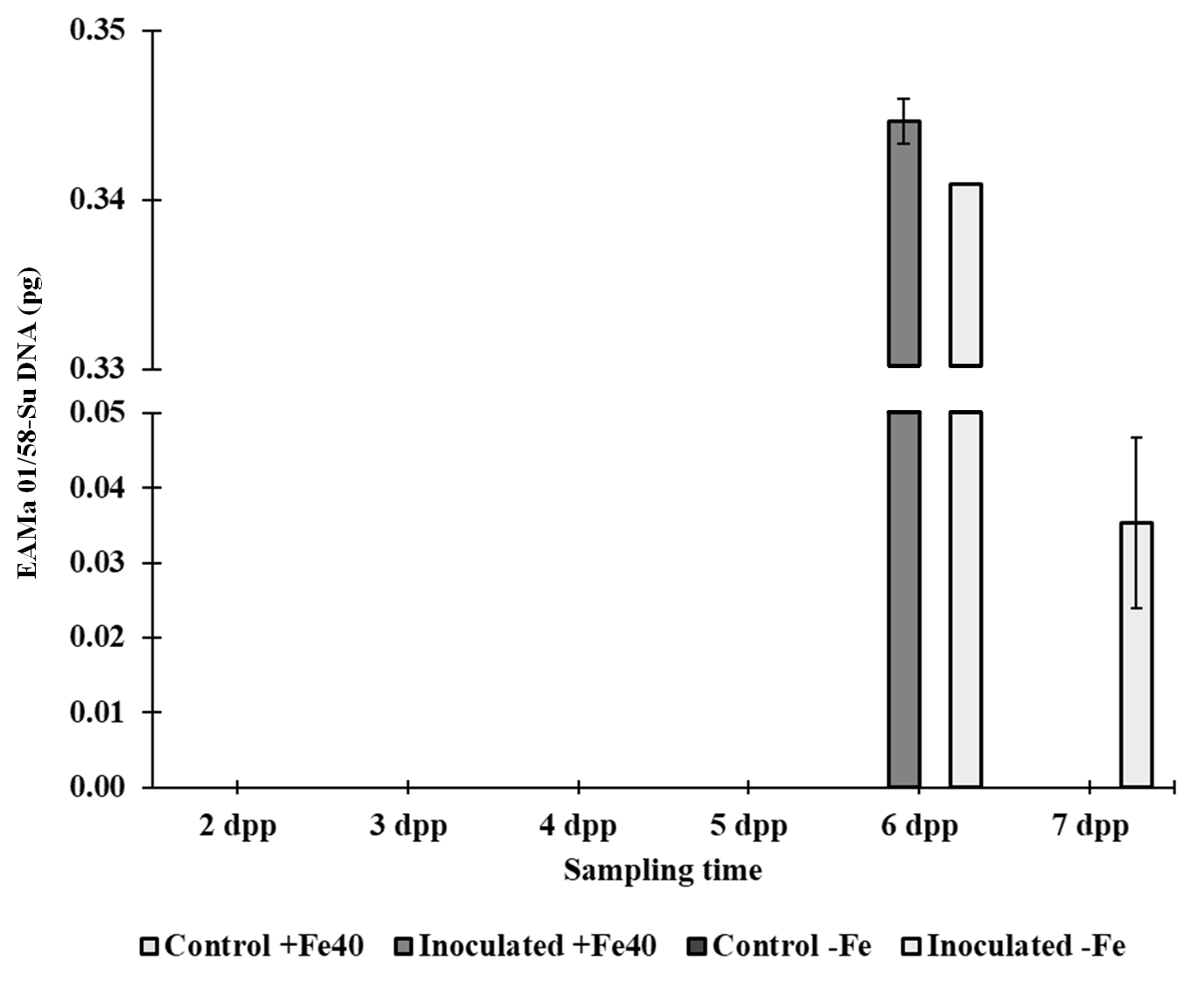


**FIG S2.** Traces of *Metarhizium brunneum* were detectable at 6 and 7 dpp on shoots of primed cucumber plants. Final concentrations was estimated by using standard curves. At 7 dpp, EAMa 01/58-Su *M. brunneum* strain was detected only in plants grown in Fe deficient conditions
